# Supplementary material for: Heritability of the HIV-1 reservoir size and decay under long-term suppressive ART
Source: Nat Commun. 2020 Nov 2;11:5542. doi: 10.1038/s41467-020-19198-7 (PMC7608612; doi:10.1038/s41467-020-19198-7)
Supplement: Supplementary file 1 — Supplementary information [file 41467_2020_19198_MOESM1_ESM.pdf]

Supplementary Information: Wan et al., Heritability of the HIV-1 reservoir size and decay under long-term suppressive ART

Table 1. (HIV-1 all subtypes): Patient characteristics of population A_0 and B_0.

		Population A_0	Population B_0
Sequenced HIV-1 genomic region		near full-length	partial *pol*
n		475	869
age at first HIV-1 DNA sample, in years (median [IQR])		42 [36,48]	41 [35,48]
ethnicity (%)	white	414 (87.16)	704 (81.01)
	non-white	61 (12.84)	165 (18.99)
sex (%)	male	385 (81.05)	673 (77.45)
	female	90 (18.95)	196 (22.55)
transmission group by sex (%)	MSM	278 (58.52)	452 (52.01)
	HET male	80 (16.84)	166 (19.1)
	HET female	71 (14.95)	151 (17.38)
	PWID male	23 (4.84)	41 (4.72)
	PWID female	8 (1.68)	22 (2.53)
	other male	7 (1.47)	19 (2.19)
	other female	8 (1.68)	18 (2.07)
time of untreated HIV-1 infection, in years (%)	<1	62 (13.05)	150 (17.26)
	1-3	69 (14.53)	106 (12.2)
	3-5	129 (27.16)	232 (26.7)
	5-7	86 (18.11)	153 (17.61)
	>7	129 (27.16)	228 (26.24)
time on ART at first HIV-1 DNA sample, in years (median [IQR])		1.5 [1.3,1.7]	1.5 [1.3-1.7]
time from ART initiation to below <50 HIV-1 RNA copies/ml, in years (median [IQR])		0.3 [0.2,0.5]	0.3 [0.2,0.5]
CD4+ cell count pre-ART/µl blood (median [IQR])		212 [130, 286]	209 [110, 293]
log 10 HIV-1 plasma RNA pre-ART/ml plasma (median [IQR])		4.9 [4.5, 5.5]	4.9 [4.4,5.4]
HIV-1 RNA (180 days after ART initiation - 1st HIV-1 DNA sample) (%)	<50 copies/ml	372 (78.32)	674 (77.56)
	viral blips	62 (13.06)	110 (12.66)
	low level viremia	38 (8.00)	80 (9.21)
	<50 copies/ml	312 (65.68)	583 (67.09)

HIV-1 RNA (1st - 3rd HIV-1 DNA sample) (%)	viral blips	121 (25.47)	214 (24.63)
	low level viremia	42 (8.84)	72 (8.29)
HIV-1 subtype available (%)		471 (99.16)	861 (99.08)
HIV-1 subtype (%) based on *pol*	B	351 (74.52)	610 (70.20)
	01_AE	36 (7.64)	65 (7.48)
	02_AG	15 (3.18)	37 (4.26)
	A	18 (3.82)	40 (4.60)
	C	19 (4.03)	35 (4.03)
	recombinant	15 (3.18)	27 (3.11)
	D	4 (0.85)	12 (1.38)
	F	4 (0.85)	12 (1.38)
	G	4 (0.85)	12 (1.38)
	Others	5 (1.06)	11 (1.27)

The time of untreated HIV-1 infection was calculated using the estimated HIV-1 infection dates. Pre-ART log10 HIV-1 RNA copies/ml plasma and pre-ART CD4+ cell count/µl blood refer to the last laboratory values available before initiation of ART. Transmission group stratified by sex indicates the self-reported route of infection (men who have sex with men (MSM), heterosexual (HET), people who inject drugs (PWID), and other (including unknown, transfusions, and perinatal transmission)). The subtypes were determined using partial *pol* Sanger sequences. ART, antiretroviral therapy.

Table 2. Number of transmission clusters extracted from phylogenies inferred from different sequences.

study population	HIV-1 genomic region	HIV-1 subtype	Size of population	Number of clusters extracted with comparable phylogenetic distance thresholds (Number of patients)			
				D1	D2	D3	D4
A_0-NGS	near full-length genome	all	475	9 (18)	21 (45)	28 (62)	41 (99)
A-NGS	near full-length genome	B	351	4 (8)	11 (23)	20 (44)	30 (74)
A-NGS	*gag*	B	349	8 (16)	14 (28)	16 (32)	28 (63)
A-NGS	*env*	B	357	9 (23)	18 (41)	24 (56)	35 (83)
A-NGS	partial *pol*	B	319	2 (4)	3 (6)	11 (22)	25 (54)
B-Sanger	partial *pol*	B	610	12 (24)	30 (65)	40 (89)	61 (143)
B_0-Sanger	partial *pol*	all	869	21 (42)	39 (83)	60 (130)	81 (192)

NGS: Next-generation sequencing; Sanger: Sanger sequencing for genotypic resistance test. For partial *pol* sequences, D1-D4 refer to 0.01,0.02,0,03,0.045 substitutions per site; for *gag* sequences, D1-D4 refer to 0.01,0.02,0.03,0.05 substitutions per site; for *env* sequences, D1-D4 refer to 0.03,0.05,0.07,0.09 substitutions per site; for viral near full-length genome sequences, D1-D4 refer to 0.04,0.05,0.06,0.09 substitutions per site.

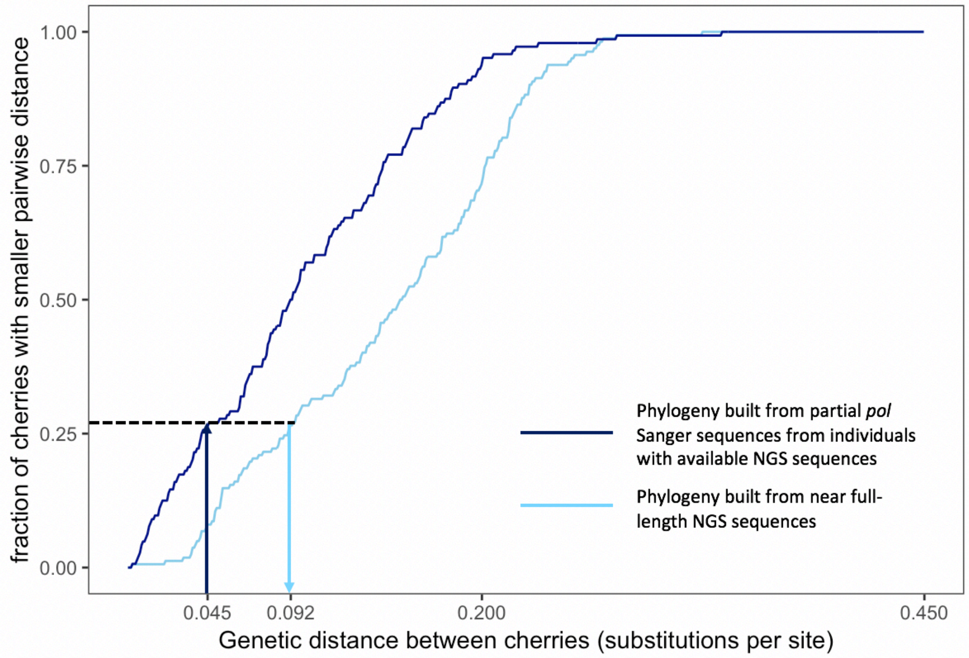

Figure 1. Explanatory plot showing how phylogenetic distance thresholds were derived for NGS *gag, env* and near full-length genome sequences. In the example, the derived genetic distance cutoff for near full-length genome NGS sequences at the comparable level of 0.045 when using partial *pol* sequences was determined such that the fraction of cherries from near full-length genome NGS phylogeny with pairwise distance lower than the cutoff was equal to the fraction of cherries from partial *pol* Sanger sequence phylogeny with pairwise distance lower than 0.045.

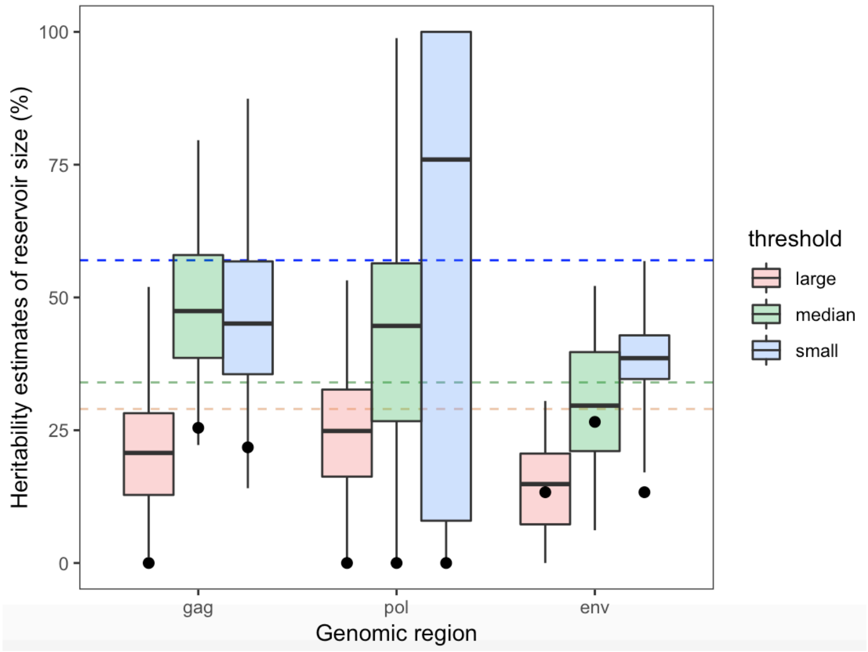

Figure 2. (HIV-1 subtype B only): Adjusted heritability estimates of HIV-1 reservoir size across the genome. Heritability was inferred with the mixed-effect model using NGS sequences of population A. The horizontal dashed lines are the viral whole-genome heritability estimates with the same method and thresholds. Small, median and large thresholds refer to the D2-D4 phylogenetic distance thresholds (Supplementary Table 2). Point estimates are shown in black dots. Boxplots represent the median, 25% and 75% quantiles of the 100 bootstrapped estimates. Whiskers represent the 95% confidence interval of the 100 bootstrapped estimates.

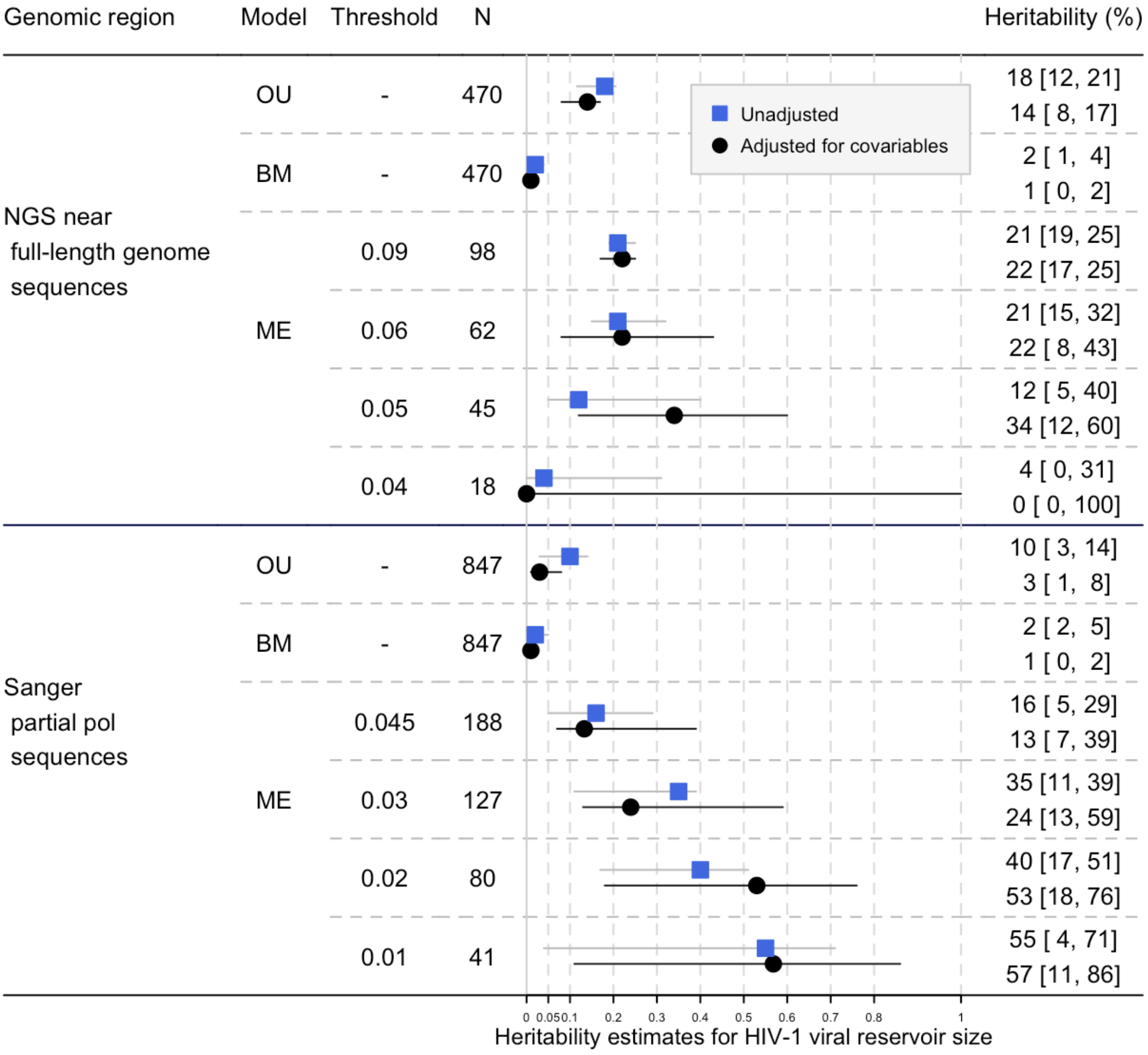

Figure 3. (HIV-1 all subtypes): Heritability estimates for HIV-1 reservoir size based on the phylogenies built from viral near full-length genome NGS sequences and partial *pol* Sanger sequences. OU: Ornstein Uhlenbeck model. BM: Brownian motion model. ME: Mixed-effect model with corresponding phylogenetic distance threshold (substitutions per site). N: Number of patients included in the analysis. Phylogenetic trees of HIV-1 all subtypes were rooted with SIV- chimpanzee references from HIV sequence database (http://www.hiv.lanl.gov). Patients with incomplete information of potential covariables were excluded. For BM and OU, all eligible patients from the tree were included while for mixed-effect model, only patients in the extracted transmission clusters were included. Black dots and black confidence intervals show the heritability estimates adjusted for covariables while blue rectangles and gray confidence intervals show the unadjusted estimates. 95% confidence intervals are shown in the square brackets. 0 heritability found using NGS sequences with the strictest threshold was due to the small sample size as the cumulative probability of zero heritability estimate was very high (30%-50% for different cutoffs) with small sample size (see Supplementary Figure 19).

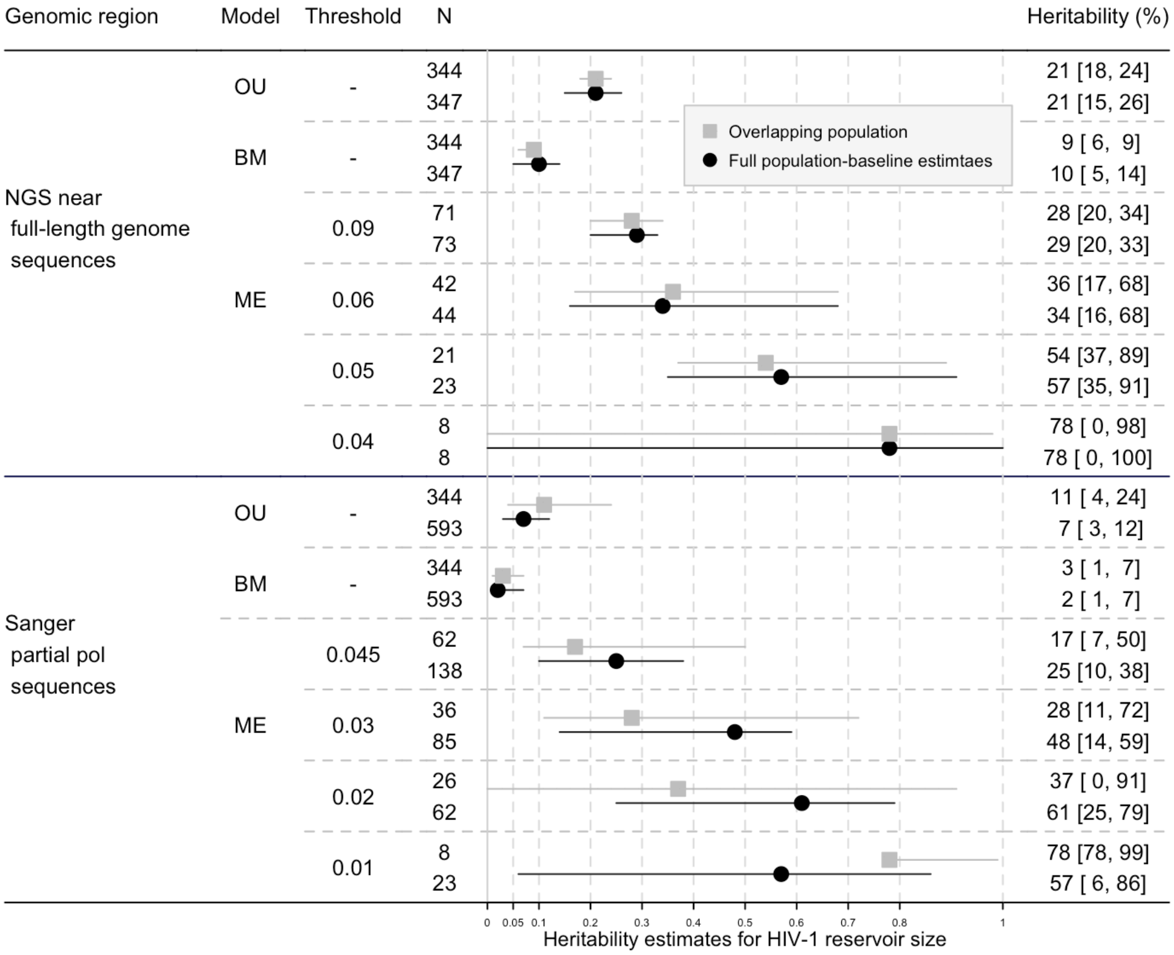

Figure 4. (HIV-1 subtype B only): Adjusted heritability estimates of HIV-1 reservoir size: from the overlapping population between population A and B, compared with the estimates from corresponding full population. Black dots ("Baseline estimates") and black confidence intervals show the estimates presented in Figure 2 of the manuscript, grey rectangles and gray confidence intervals show the estimates from the overlapping population. 95% confidence intervals are shown in the square brackets.

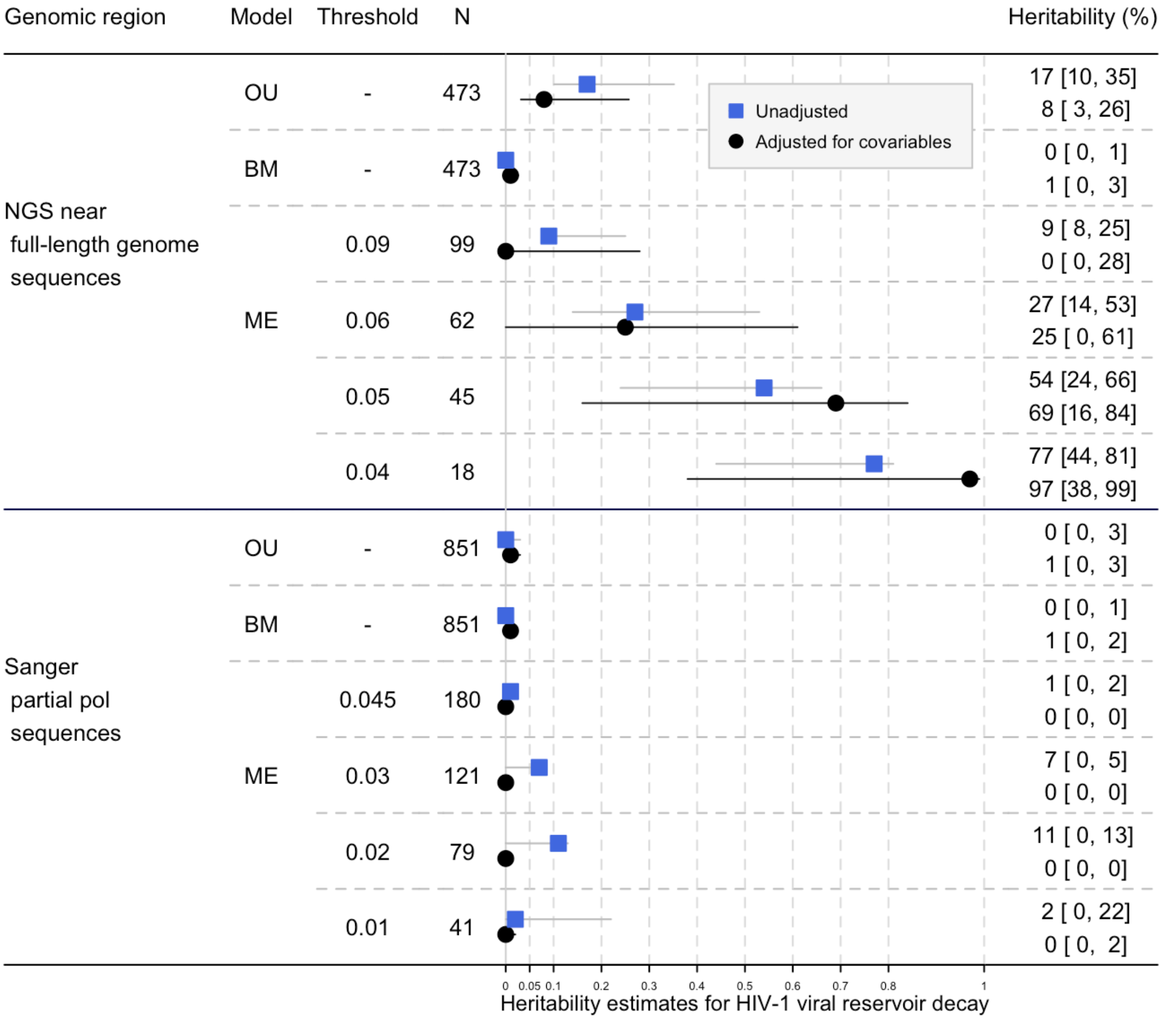

Figure 5. (HIV-1 all subtypes): Heritability estimates for HIV-1 reservoir decay slope based on the phylogenies built from viral near full-length genome NGS sequences and partial *pol* Sanger sequences. OU: Ornstein Uhlenbeck model. BM: Brownian motion model. ME: Mixed-effect model with corresponding phylogenetic distance threshold (substitutions per site). N: Number of patients included in the analysis. Phylogenetic trees of HIV-1 all subtypes were rooted with SIV-chimpanzee references from HIV sequence database (http://www.hiv.lanl.gov). Patients with incomplete information of potential covariables were excluded. Patients with incomplete information of potential covariables were excluded. For OU and BM, all eligible patients from the tree were included while for mixed-effect model, only patients in the extracted transmission clusters were included. Black dots and black confidence intervals show the heritability estimates adjusted for covariables while blue rectangles and gray confidence intervals show the unadjusted estimates. 95% confidence interval are shown in the square brackets.

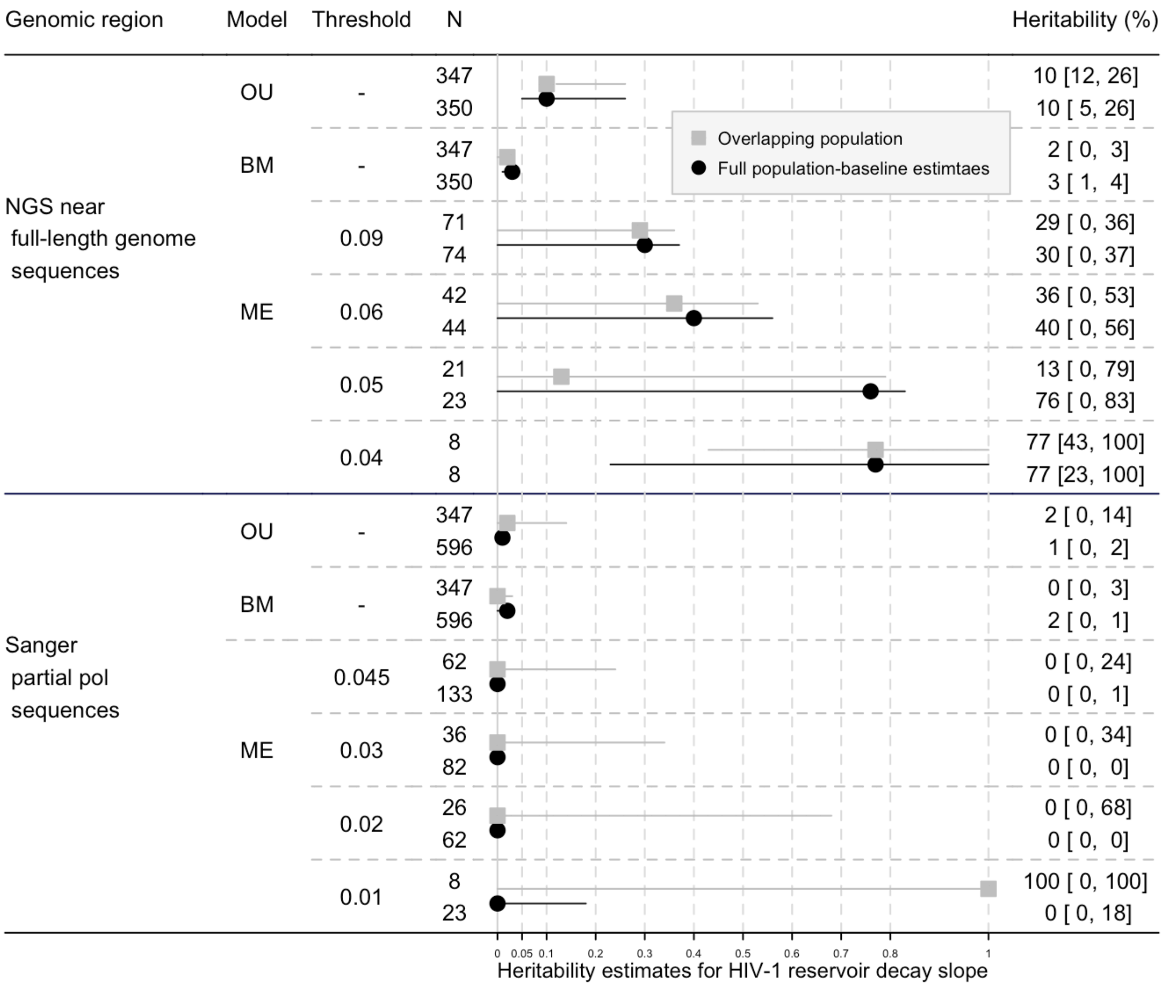

Figure 6. (HIV-1 subtype B only): Adjusted heritability estimates of HIV-1 reservoir decay slope: from the overlapping population between population A and B, compared with the estimates from corresponding full population. Black dots (“Baseline estimates”) and black confidence intervals show the estimates presented in Figure 3 of the manuscript, grey rectangles and gray confidence intervals show the estimates from the overlapping population. 95% confidence intervals are shown in the square brackets.

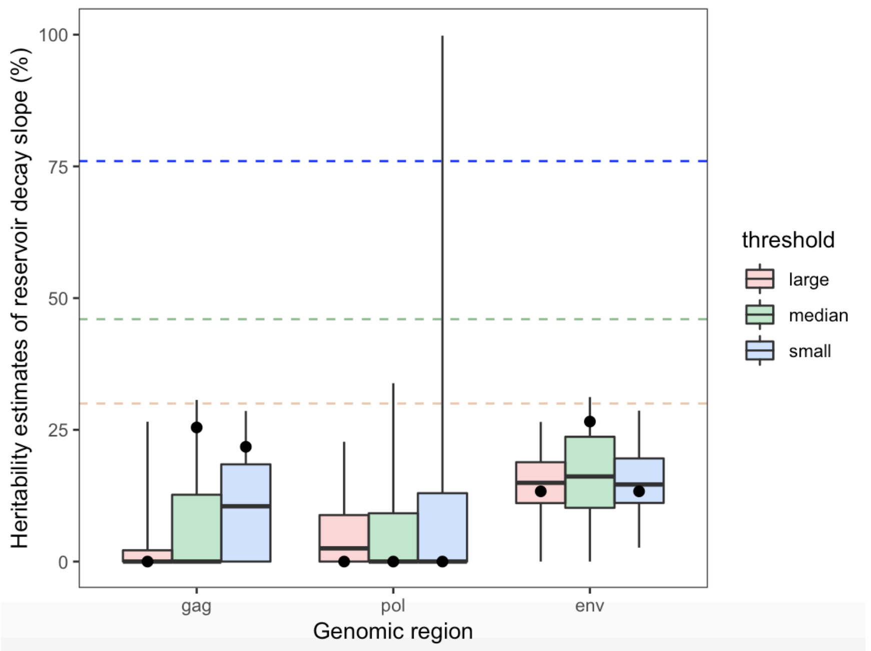

Figure 7. (HIV-1 subtype B only): Adjusted heritability estimates of HIV-1 reservoir decay slope across the genome. Heritability was inferred with the mixed-effect model using NGS sequences of population A. The horizontal dashed lines are the whole-genome heritability estimates with the same method and thresholds. Small, median and large thresholds refer to the D2-D4 phylogenetic distance thresholds (Supplementary Table 2). Point estimates are shown in black dots. Boxplots represent the median, 25% and 75% quantiles of the 100 bootstrapped estimates. Whiskers represent the 95% confidence interval of the 100 bootstrapped estimates.

Table 3. (HIV-1 subtype B only): Goodness of fit test for estimating heritability of HIV-1 reservoir size.

Sequences	Method	Threshold	N	Unadjusted				Adjusted			
				loglik	AIC	BIC	logPost	loglik	AIC	BIC	logPost
NGS whole genome	null model	-	347	-257.53	519.05	526.75		-227.80	489.61	555.05	
	OU	-	347	-251.37	512.91	531.98	-260.06	-226.92	464.02	570.83	-235.27
	BM	-	347	-252.96	511.98	523.46	-254.86	-228.34	462.75	561.97	-229.98
	mixed-effect model	0.09	347	-252.04	510.09	521.63	-	-227.78	491.56	560.85	-
		0.07	347	-253.14	512.29	523.83	-	-228.30	492.60	561.89	-
		0.06	347	-253.21	512.42	523.97	-	-229.00	494.00	563.29	-
		0.05	347	-253.61	513.21	524.76	-	-228.96	493.93	563.22	-
Sanger partial pol	null model	-	593	-428.88	861.77	870.54		-375.53	785.05	859.60	
	OU	-	593	-410.92	831.95	853.78	-420.51	-374.19	758.49	876.09	-382.48
	BM	-	593	-414.28	834.60	847.72	-416.15	-375.53	757.09	865.99	-377.19
	mixed-effect model	0.045	593	-412.31	830.61	843.77	-	-374.04	784.09	863.02	-
		0.03	593	-411.42	828.84	842.00	-	-373.46	782.92	861.86	-
		0.02	593	-411.72	829.44	842.59	-	-373.23	782.46	861.39	-
		0.01	593	-410.33	826.65	839.81	-	-372.76	781.53	860.46	-

Largest loglik and lowest AIC were marked red. Detailed description of methods can be found in our Supplementary Methods.

Table 4. (HIV-1 subtype B only) Goodness of fit test for estimating heritability of HIV-1 reservoir decay slope.

Sequences	Method	Threshold	N	Unadjusted				Adjusted			
				loglik	AIC	BIC	logPost	loglik	AIC	BIC	logPost
NGS whole genome	null model	-	350	-257.53	519.05	526.77		354.77	-683.54	-633.39	
	OU	-	350	275.18	-540.19	-521.08	270.56	356.83	-703.49	-619.94	353.72
	BM	-	350	272.85	-539.64	-528.13	274.04	353.28	-700.48	-624.54	354.89
	mixed-effect model	0.09	350	273.23	-540.46	-528.88	-	354.77	-681.54	-627.53	-
		0.07	350	273.07	-540.14	-528.57	-	354.22	-680.43	-626.42	-
		0.06	350	274.56	-543.11	-531.54	-	355.37	-682.75	-628.74	-
		0.05	350	274.43	-542.85	-531.28	-	354.82	-681.63	-627.62	-
Sanger partial pol	null model	-	596	487.18	-970.36	-961.58		611.66	-1197.32	-1140.24	
	OU	-	596	482.00	-953.90	-932.05	479.16	609.01	-1207.92	-1115.78	606.42
	BM	-	596	481.64	-957.25	-944.12	482.75	608.70	-1211.36	-1127.94	610.17
	mixed-effect model	0.045	596	483.57	-961.14	-947.97	-	611.66	-1195.32	-1133.85	-
		0.03	596	483.57	-961.14	-947.97	-	611.66	-1195.32	-1133.85	-
		0.02	596	483.57	-961.14	-947.97	-	611.66	-1195.32	-1133.85	-
		0.01	596	483.57	-961.14	-947.97	-	611.66	-1195.32	-1133.85	-

Largest loglik and lowest AIC were marked red. Detailed description of methods can be found in our Supplementary Methods.

Table 5. (HIV-1 subtype B only): p value from the significance test of the estimated heritability.

Sequences	Method	N	Reservoir size		Reservoir decay slope	
			Unadjusted	Adjusted	Unadjusted	Adjusted
NGS near full-length genome	BM	350	0.000**	0.000**	0.000**	0.000**
	OU	350	0.000**	0.000**	0.000**	0.000**
	ME-0.09	74	0.056	0.048*	0.029*	0.428
	ME-0.06	44	0.253	0.168	0.080	0.096
	ME-0.05	23	0.283	0.181	0.157	0.177
	ME-0.04	8	0.668	1.000	0.020*	0.521
Sanger partial *pol*	BM	596	0.000**	0.000**	0.000**	0.000**
	OU	596	0.000**	0.000**	0.000**	0.000**
	ME-0.045	138	0.046*	0.057	1.000	1.000
	ME-0.03	85	0.014*	0.005**	1.000	1.000
	ME-0.02	62	0.023*	0.005**	1.000	1.000
	ME-0.01	23	0.006**	0.161	1.000	1.000

For mixed-effect model, p value was achieved from the likelihood ratio test of the random effects (transmission clusters), using ranova function from the R package lmerTest v 3.1-0[1]. For BM/OU model, p value was achieved from the likelihood ratio test of the model fitting with corresponding white-noise model where all the phenotypic variance was explained by a Gaussian model, using lrtest from the R package lmtest v 0.9-36[2]. **: p<0.01, *:p<0.05.

Table 6. (HIV-1 all subtypes): p value from the significance test of the estimated heritability.

Sequences	Method	N	Reservoir size		Reservoir decay slope	
			Unadjusted	Adjusted	Unadjusted	Adjusted
NGS near full-length genome	BM	473	0.000**	0.000**	0.000**	0.000**
	OU	473	0.000**	0.000**	0.000**	0.000**
	ME-0.09	111	0.022*	0.029*	0.425	1.000
	ME-0.06	69	0.151	0.184	0.044*	0.237
	ME-0.05	48	0.571	0.206	0.006**	0.049*
	ME-0.04	20	0.910	1.00	0.005**	0.025*
Sanger partial *pol*	BM	851	0.000**	0.000**	0.000**	0.000**
	OU	851	0.000**	0.000**	0.000**	0.000**
	ME-0.045	188	0.066	0.185	1.000	1.000
	ME-0.03	127	0.003**	0.071	0.599	1.000
	ME-0.02	80	0.006**	0.004**	0.451	1.000
	ME-0.01	41	0.007**	0.02*	0.933	1.000

For mixed-effect model, p value was achieved from the likelihood ratio test of the random effects (transmission clusters), using ranova function from the R package lmerTest v 3.1-0[1]. For BM/OU model, p value was achieved from the likelihood ratio test of the model fitting with corresponding white-noise model where all the phenotypic variance was explained by a Gaussian model, using lrtest from the R package lmtest v 0.9-36[2]. **: p<0.01, *:p<0.05.

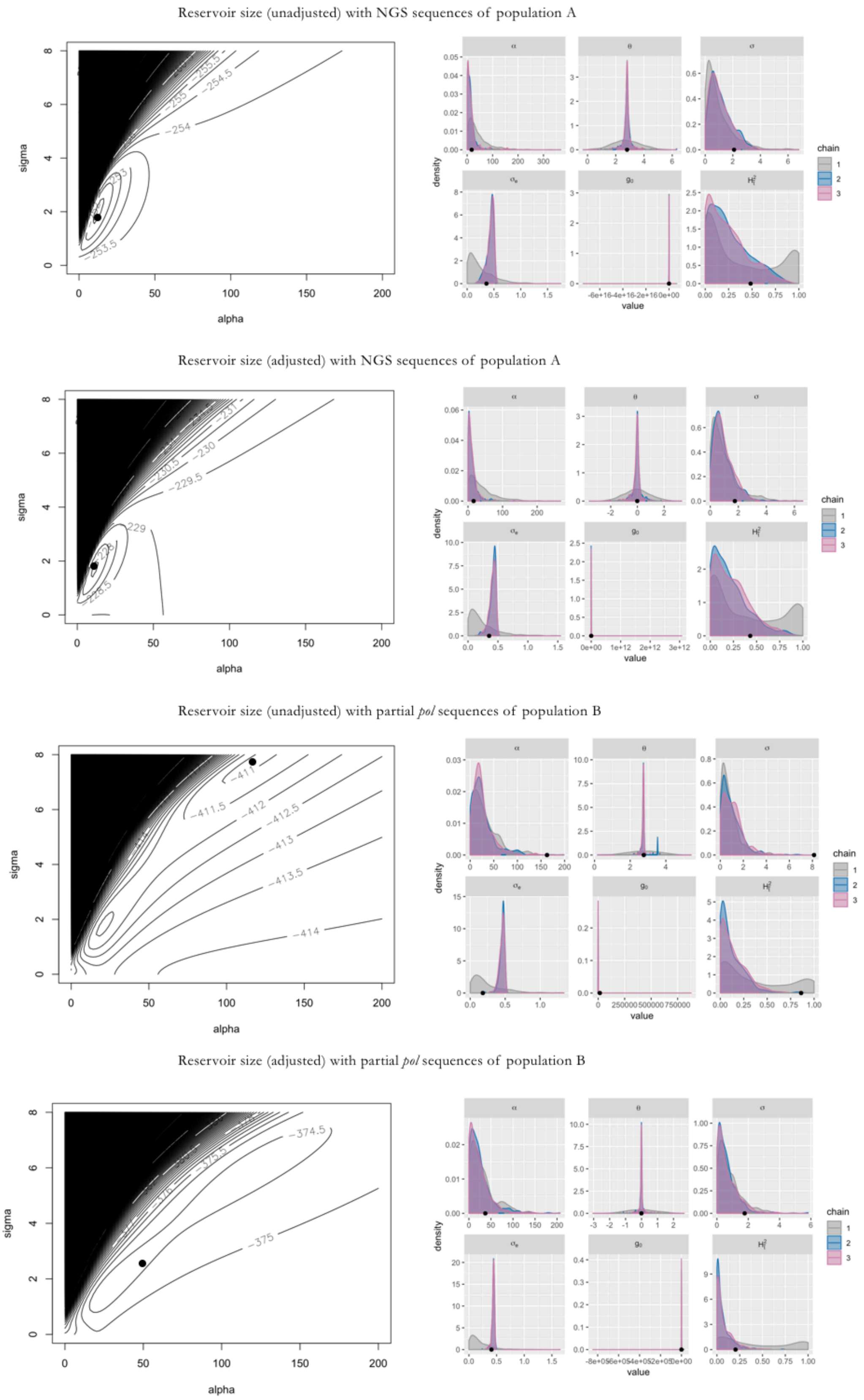

Figure 8. Diagnostic plots for heritability estimation of HIV-1 reservoir size with Ornstein Uhlenbeck model. For each set of plots, the left plot shows the likelihood surface of OU optimization based on parameters alpha and sigma and the right plot shows the MCMC univariate density plots from the POUMM package. Black dots in both plots show the maximum likelihood of parameters.

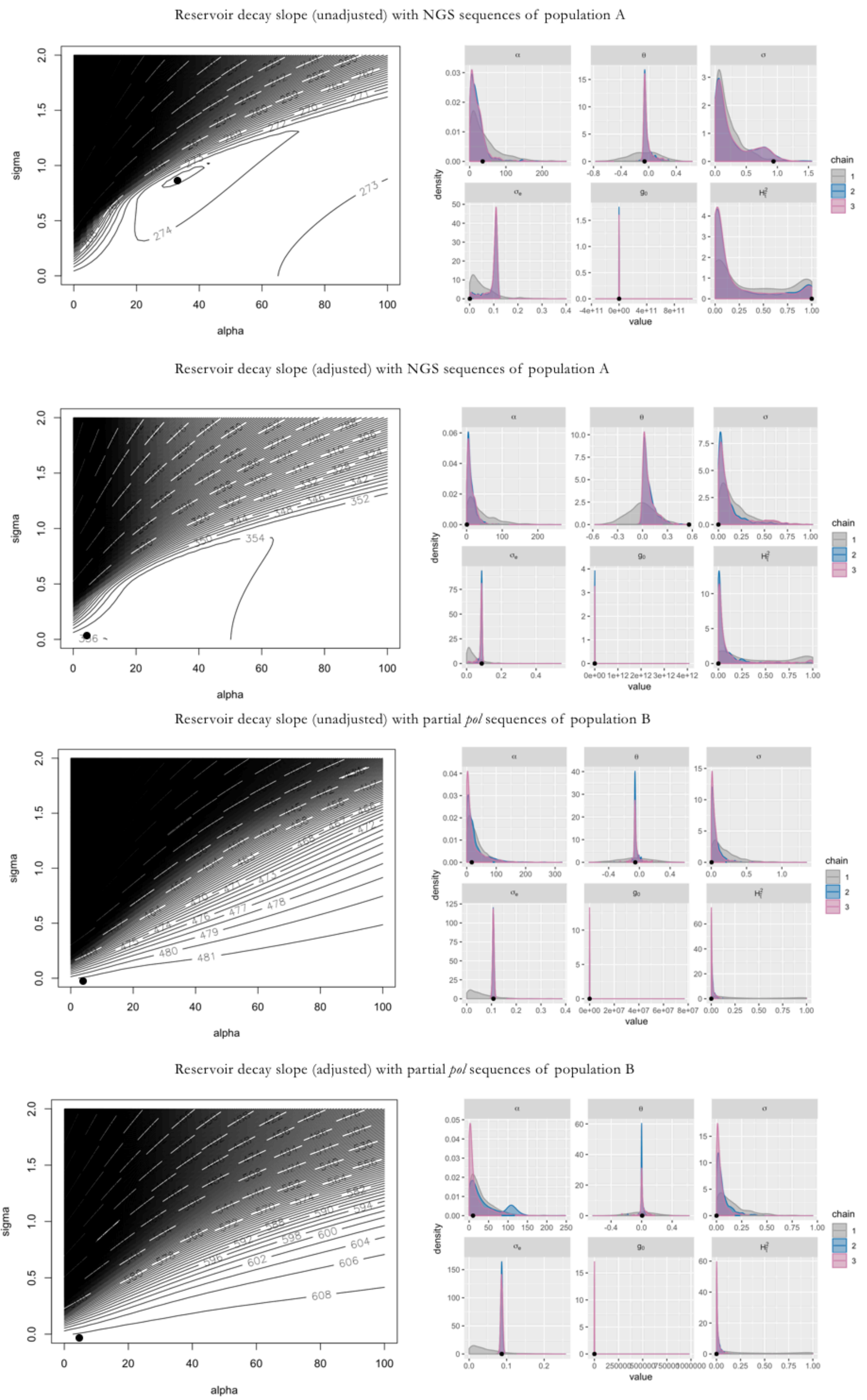

Figure 9. Diagnostic plots for heritability estimation of HIV-1 reservoir decay slope with Ornstein Uhlenbeck model. For each set of plots, the left plot shows the likelihood surface of OU optimization based on parameters alpha and sigma and the right plot shows the MCMC univariate density plots from the POUMM package. Black dots in both plots show the maximum likelihood of parameters.

Supplementary Discussion: Interpreting the heritability estimates from phylogenetic mixed model

Phylogenetic mixed models are widely used to estimate heritability from a phylogenetic tree, based on the assumptions that the trait evolved along the tree according to Brownian motion (BM) or Ornstein Uhlenbeck (OU) model respectively. We applied this method in our analysis to estimate the heritability of the HIV-1 reservoir size and decay slope under long-term ART. As a pre-fitting test, our data shows a good trend towards uni-modality and normal distribution of trait values according to root-to-tip distance (Supplementary Figure 10).

Heritability of HIV-1 reservoir size

Using the R package POUMM[3], we estimated the unadjusted heritability to be 12% [6%, 25%] fitted with OU model and 3% [2%, 10%] fitted with BM model using phylogeny inferred from partial *pol* Sanger sequences in population B. The OU model, which can be seen as an extension of the BM model with an addition of a stabilizing selection towards the optimal trait value, provided a significantly better fit to the data (Likelihood ratio test: P = 0.03). Thus, the 12% estimate yielded from OU model were thought to have relatively more statistical support.

The optimal trait value θ estimated from OU model was 2.68 [2.64 – 2.84] log10 total HIV-1 DNA copies/1 million genomic equivalents (copies/mge), which was close to the mean HIV-1 reservoir size in our study population (2.76 log10 total DNA copies/mge) (Supplementary Table 7). The optimized selection strength α was 25.08 [10.54 – 154.98]. The phylogenetic half-life, which describes the time to move halfway from the ancestral state to the optimum[4] was 0.028 [0.004, 0.066]. This was lower than the minimum branch length 0.067, thus indicating that the selection towards the current mean trait value is strong. Similar conclusions were found also for the HIV-1 set-point viral load[5]. However, as discussed by Mitov and Stadler[3], the parameter α has a dual interpretation as both, a rate of convergence of the mean towards the long-term optimum, and a rate of decorrelation between the trait values in phylogenetic pairs. Hence, it is possible to infer high values for the parameter α, even in cases of neutral evolution (shown in toy model simulations[3]). For this reason and given the observed overlap between the posterior and the prior distributions for the parameter α (Supplementary Figure 8-9), our further interpretation of high values for α as evidence for stabilizing selection is only hypothetical.

After adjustment, heritability estimates with OU and BM model decreased to 7% [3%, 12%] and 2% [1%, 7%] respectively. However, the OU model didn't provide a significantly better fit to the data compared with BM model (Likelihood ratio test: P=0.298). The optimal trait value θ and selection strength α estimated from OU model increased to 2.89 (2.84 – 3.03) total HIV-1 DNA copies/mge and 24.51 [10.26, 175.75], respectively after adjustment. The phylogenetic half-life was 0.028 [0.004, 0.067].

Using viral near full-length genome NGS sequences of population A, the phylogenetic mixed model assuming a trait evolution according to OU and BM model yielded higher adjusted heritability estimates of OU: 21% [15%, 26%] and BM: 10% [5%, 14%]. However, the OU model didn't provide a statistically significant better fit to this sub-dataset compared to BM model for both unadjusted and

adjusted trait values (Likelihood ratio test: P=0.204 for unadjusted and P=0.242 for adjusted reservoir size).

Heritability of HIV-1 reservoir decay slope

Applying OU model, the adjusted heritability of HIV-1 reservoir decay slope using viral near full-length genome sequences were significantly larger than zero, less so for heritability estimates derived with phylogeny built from partial *pol* sequences. BM model yielded estimates close to zero for both near full-length genome sequences and partial *pol* sequences. When estimating the heritability of reservoir decay slope, OU model didn't provide significantly better fit compared with BM model for all cases. The optimal trait value estimated from OU model was -0.03 for unadjusted decay slope, which was similar with the population mean (Supplementary Table 7). The high α optimized from OU model for decay slope could also suggest a strong stabilizing selection strength around the optimal value.

Discussion

The above heritability estimates based on OU and BM model were fitted with the Bayesian inference in the R package POUMM. In a sensitivity analysis (Supplementary Table 8-9), we found that applying maximum likelihood optimization yielded more unstable heritability estimates compared with Bayesian inference. In some cases, maximum likelihood estimates reached 100%. The unstable estimates can be explained by the flat likelihood surface of model fitting. Restricting the upper limit of α reduced the chance of overestimating heritability estimates. The upper limit of 10 was used in Blanquart et al[6] based on the BEEHIVE phylogeny. Though our dataset is more densely sampled which would allow for a higher α upper limit, we applied the same upper limit of 10 as it yielded comparable estimates with Bayesian inference in our study. However, it still remains unclear about the biological meanings of α in specific settings and especially what would be the proper range for a realistic α.

Across different model implementations and study populations, a relatively large α was inferred for both reservoir size and decay (Supplementary Table 7-9). Whether or not the estimated large α actually indicates a real biological signal of stabilizing selection towards the optimal value remains unclear. Other HIV-related traits such as set-point viral load were confirmed to undergo such selection process[5]. However, to our knowledge, no studies have been done until now concerning the evolution process of reservoir-related traits under treatment on population levels. So, further evidence would be required for better interpreting the parameter estimates from phylogenetic mixed models.

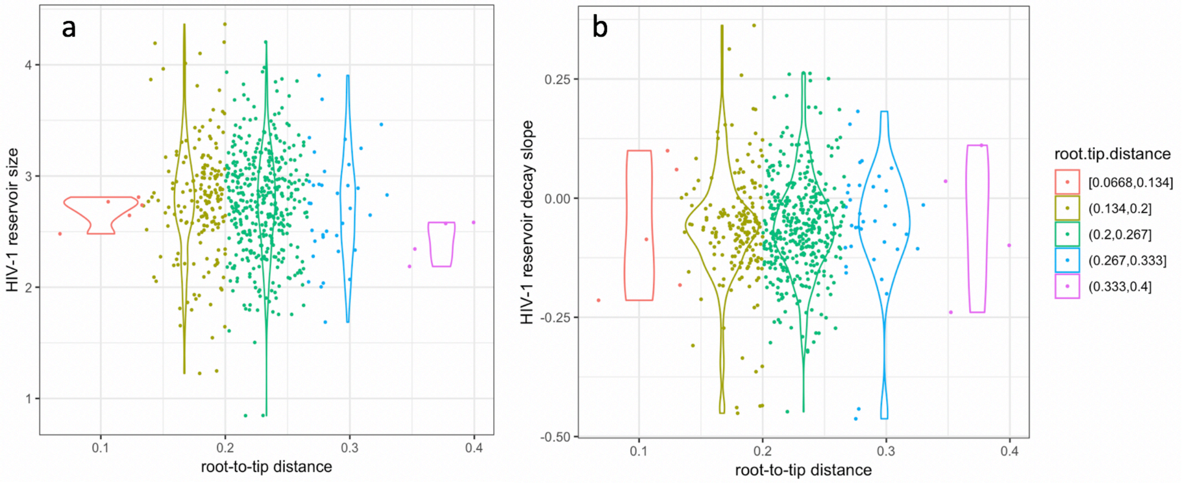

Figure 10. (HIV-1 subtype B only): Distributions of the trait-values grouped by root-tip distance. (a). HIV-1 reservoir size in log10 total HIV-1 DNA copies/1 million genomic equivalents. (b) HIV-1 reservoir decay slope.

Table 7. (HIV-1 subtype B only): Estimates of the OU model parameters for reservoir size and decay slope with Bayesian inference from the R package POUMM.

	Genomic region	Adjustment	alpha	theta	Population mean
Reservoir size	partial *pol*	unadjusted	25.08 [10.54, 154.98]	2.68 [2.64, 2.84]	2.76
	partial *pol*	adjusted	24.51 [10.26, 175.75]	2.89 [2.84, 3.03]	2.76
	near full-length genome	unadjusted	12.99 [9.70, 101.70]	2.78 [2.65, 2.94]	2.81
	near full-length genome	adjusted	13.38 [9.96, 24.16]	2.88 [2.89, 3.09]	2.81
Decay slope	partial *pol*	unadjusted	17.80 [11.66, 258.56]	-0.03 [-0.10, -0.00]	-0.06
	partial *pol*	adjusted	34.90 [9.85, 258.79]	0.31 [0.27, 0.36]	-0.06
	near full-length genome	unadjusted	20.63 [17.58, 192.30]	-0.01 [-0.07, 0.01]	-0.07
	near full-length genome	adjusted	11.35 [8.84, 29.49]	0.39 [0.32, 0.42]	-0.07

Alpha: selection strength; theta: optimal trait value; unit of optimal trait value and population mean for reservoir size: log10 total HIV-1 DNA copies/1 million genomic equivalents. 95% confidence intervals are shown in the square brackets.

Table 8. (HIV-1 subtype B only): Comparison of heritability estimates for HIV-1 viral reservoir size using maximum likelihood optimization with two implementations.

Study population	Genomic region	Subtype	Model	POUMM package		Blanquart et al, 2017	
				Heritability (%)*	alpha*	Heritability (%)*	alpha*
A	near full-length genome	B	OU-no adjustment	49 [2.1, 53.2]	17.9 [16.6, 366.9]	31.7 [26.4, 37.9]	10 [10, 10]
A	near full-length genome	B	OU-adjustment	43.5 [43.5, 54.9]	16.2 [16.2, 24.6]	29 [26, 37.5]	10 [9.2, 10]
A	near full-length genome	B	BM-no adjustment	12.4 [0.3, 17.1]		11.8 [6.6, 17]	
A	near full-length genome	B	BM-adjustment	11 [0.3, 16.8]		10.9 [9, 18.9]	
B	partial *pol*	B	OU-no adjustment	86 [16, 88]	154.6 [24.9, 415.7]	11 [3, 7]	10 [0, 10]
B	partial *pol*	B	OU-adjustment	18 [7, 67]	34 [19.7, 437.4]	4 [0, 11]	10 [0, 10]
B	partial *pol*	B	BM-no adjustment	0 [0, 10]		0 [0, 10]	
B	partial *pol*	B	BM-adjustment	0 [0, 8]		0 [0, 7]	

*95% confidence intervals are shown in the square brackets.

Table 9. (HIV-1 subtype B only): Comparison of heritability estimates for HIV-1 viral reservoir decay slope using maximum likelihood optimization with two implementations.

Study population	Genomic region	Subtype	Model	POUMM package		Blanquart et al, 2017	
				Heritability (%)*	alpha*	Heritability (%)*	alpha*
A	near full-length genome	B	OU-no adjustment	100 [95.5, 100]	35.8 [34.5, 380.3]	0.2 [0, 1.3]	10 [0, 10]
A	near full-length genome	B	OU-adjustment	2.3 [1.3, 90.8]	1.3 [0.9, 53.9]	0.1 [0, 1]	0 [0, 10]
A	near full-length genome	B	BM-no adjustment	0.3 [0.3, 0.3]		0 [0, 0]	
A	near full-length genome	B	BM-adjustment	0.3 [0.3, 0.3]		0 [0, 0]	
B	partial *pol*	B	OU-no adjustment	0 [0, 51]	14.3 [14.3, 517]	0 [0, 1]	10 [0, 10]
B	partial *pol*	B	OU-adjustment	0 [0, 9]	11 [11, 515.9]	0 [0, 1]	10 [0, 10]
B	partial *pol*	B	BM-no adjustment	0 [0, 0]		0 [0, 0]	
B	partial *pol*	B	BM-adjustment	0 [0, 0]		0 [0, 0]	

*95% confidence intervals are shown in the square brackets.

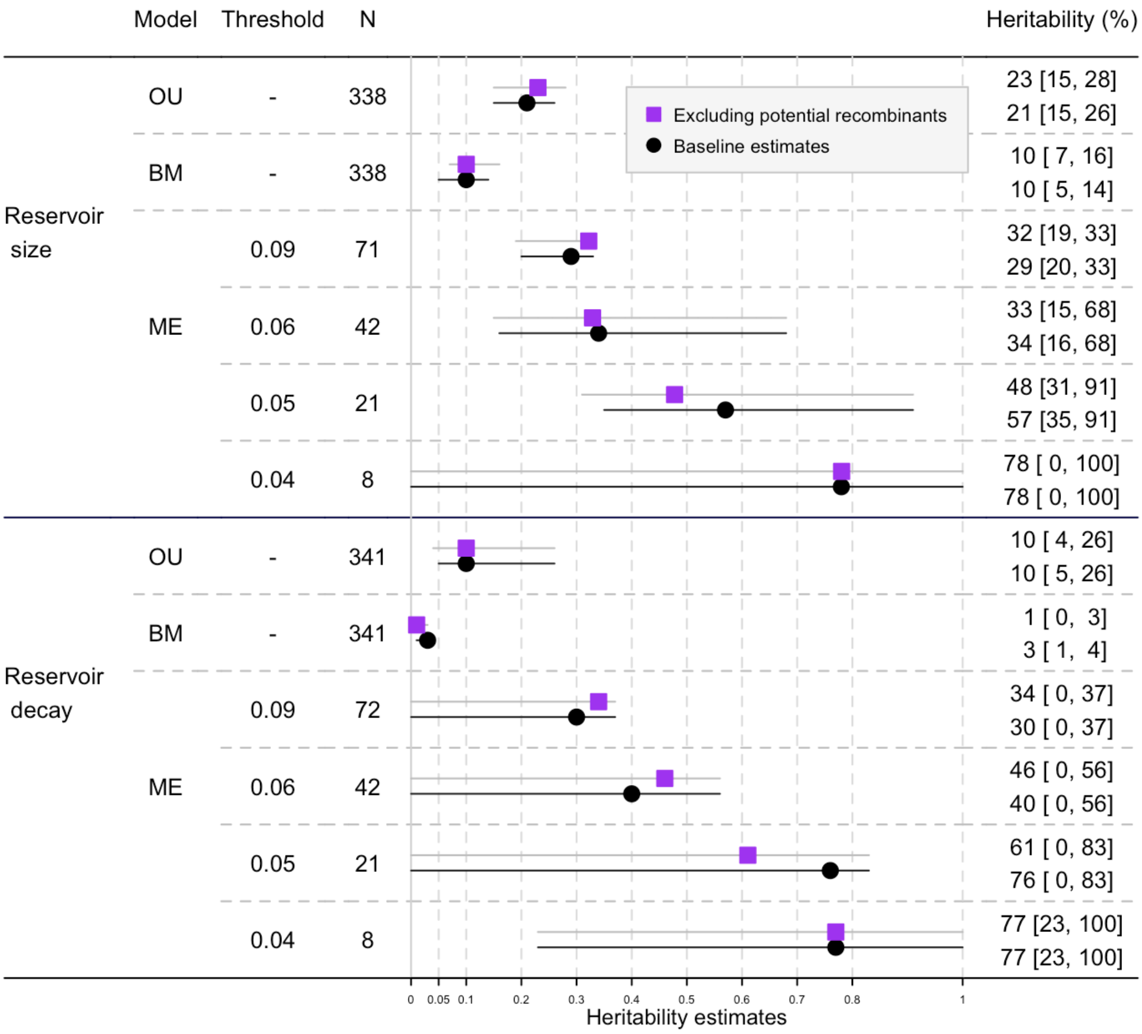

Figure 11. (HIV-1 subtype B only): Heritability estimates adjusted for covariables using the phylogeny built from near full-length HIV-1 genome NGS sequences: Sensitivity analysis excluding potential recombinants identified by Comet[7]. Black dots ("Baseline estimates") and black confidence intervals show the estimates presented in Figure 2 and 3 of the manuscript, violet rectangles and gray confidence intervals show the according estimates after exclusion of recombinants identified by Comet[7]. 95% confidence intervals are shown in square brackets.

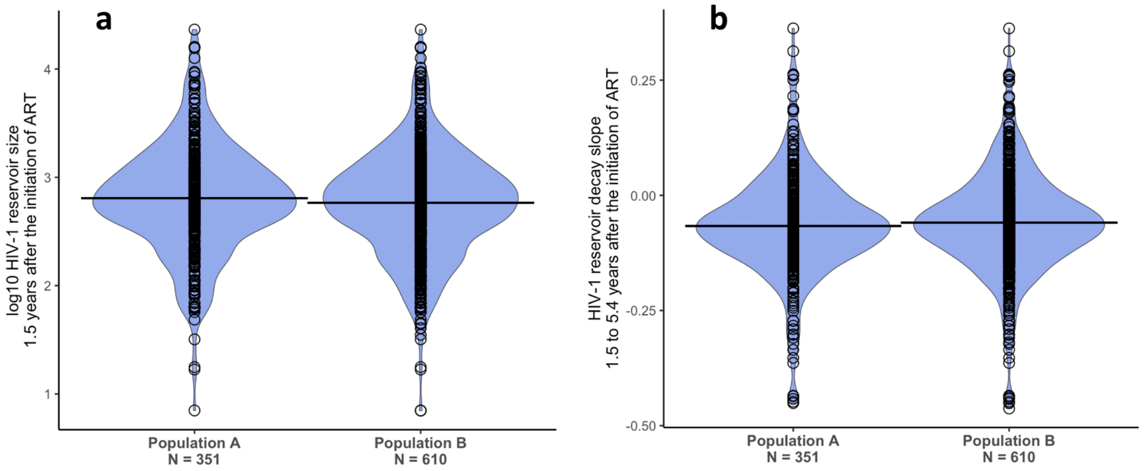

Figure 12. Violin plots of HIV-1 reservoir size (subplot a) and decay slope (subplot b) in population A and population B. N represents the number of individuals in the two population. The individual observations are shown in small circles and the average is shown as black line.

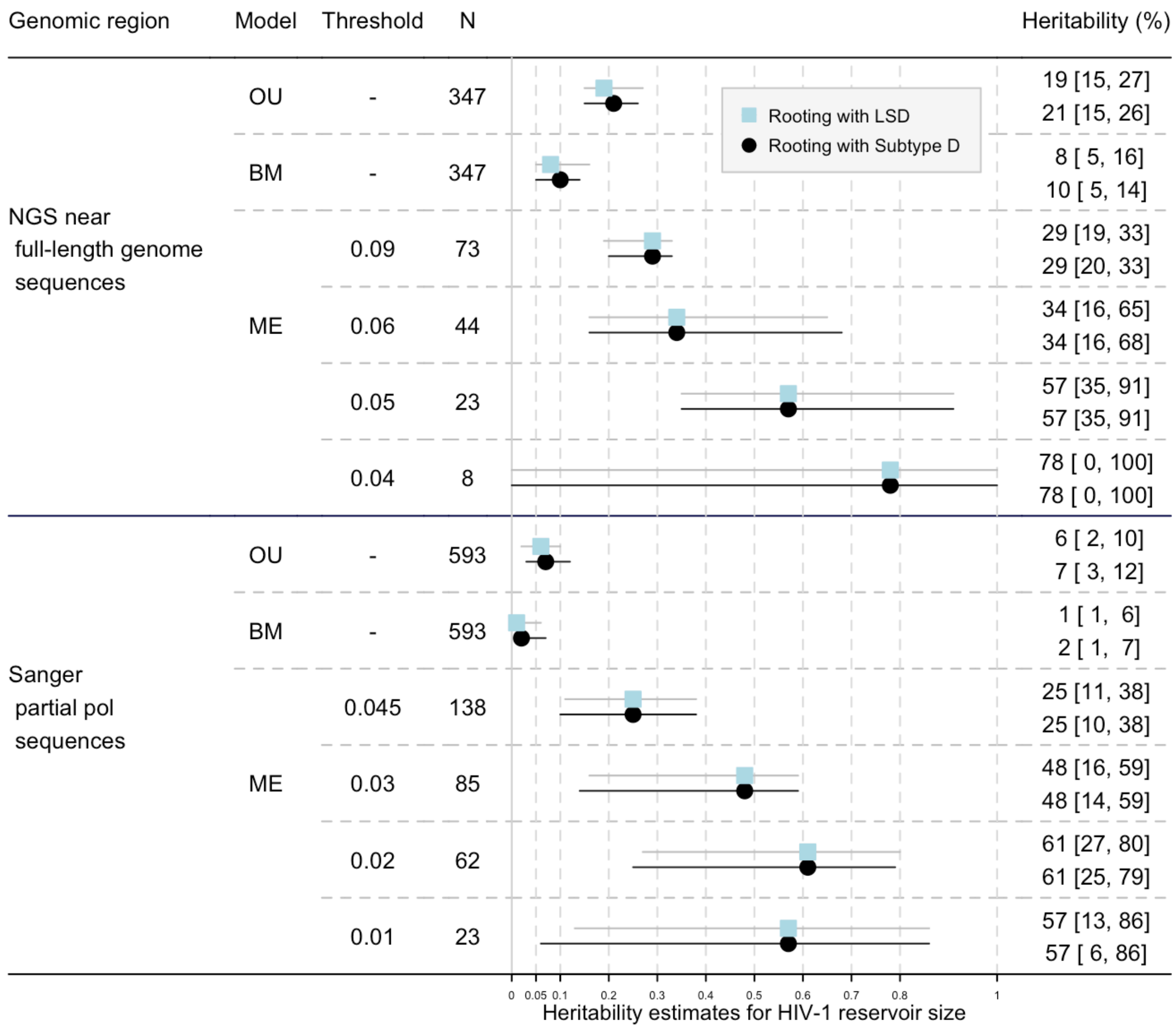

Figure 13. (HIV-1 subtype B only): Heritability estimates adjusted for covariables using different rooting methods for the HIV-1 reservoir size 1.5 years after initiation of ART. Black dots ("Rooting with HIV-1 subtype D") and black confidence intervals show the estimates presented in Figure 2 of the manuscript. Light blue rectangles ("Rooting with LSD") and grey confidence intervals show the estimates from the phylogeny rooted with the root position found by LSD. The rate of evolution and tMRCA estimated by LSD can be found in Supplementary Table 10. 95% confidence intervals are shown in square brackets.

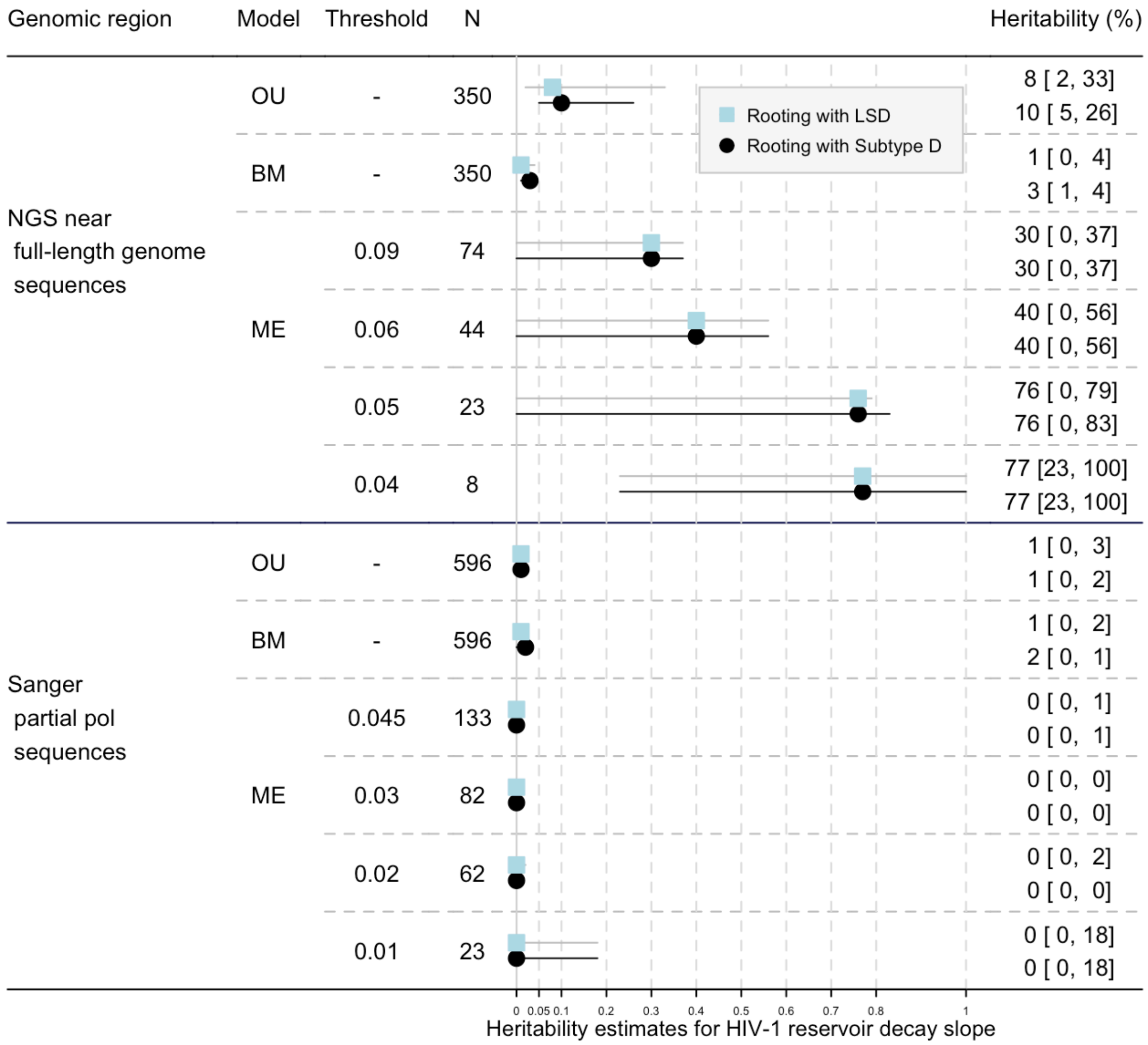

Figure 14. (HIV-1 subtype B only): Heritability estimates adjusted for covariables using different rooting methods for the HIV-1 reservoir decay slope 1.5-5.4 years after initiation of ART. Black dots ("Rooting with subtype D") and black confidence intervals show the estimates presented in Figure 3 of the manuscript. Light blue rectangles ("Rooting with LSD") and grey confidence intervals show the estimates from the phylogeny rooted with the root position found by LSD. The rate of evolution and tMRCA estimated by LSD can be found in Supplementary Table 10. 95% confidence intervals are shown in square brackets.

Table 10. Estimated rate of evolution and tMRCA with the HIV-1 NGS near full-length genome and Sanger partial *pol* genome dataset, using LSD-0.2[8] (both based on HIV-1 subtype B only).

	Estimated rate of evolution (substitutions per site per year)*	tMRCA (calender year)*
NGS near full-length genome	0.003439 [0.002849, 0.003886]	1953 [1944, 1962]
Sanger partial *pol* genome	0.003198 [0.002088, 0.003306]	1967 [1942, 1968]

*95% confidence intervals are shown in the square brackets. While the estimated tMRCA is earlier when based on near full viral genomes than when based on *pol*, it should be noted that the confidence intervals are broad and overlapping.

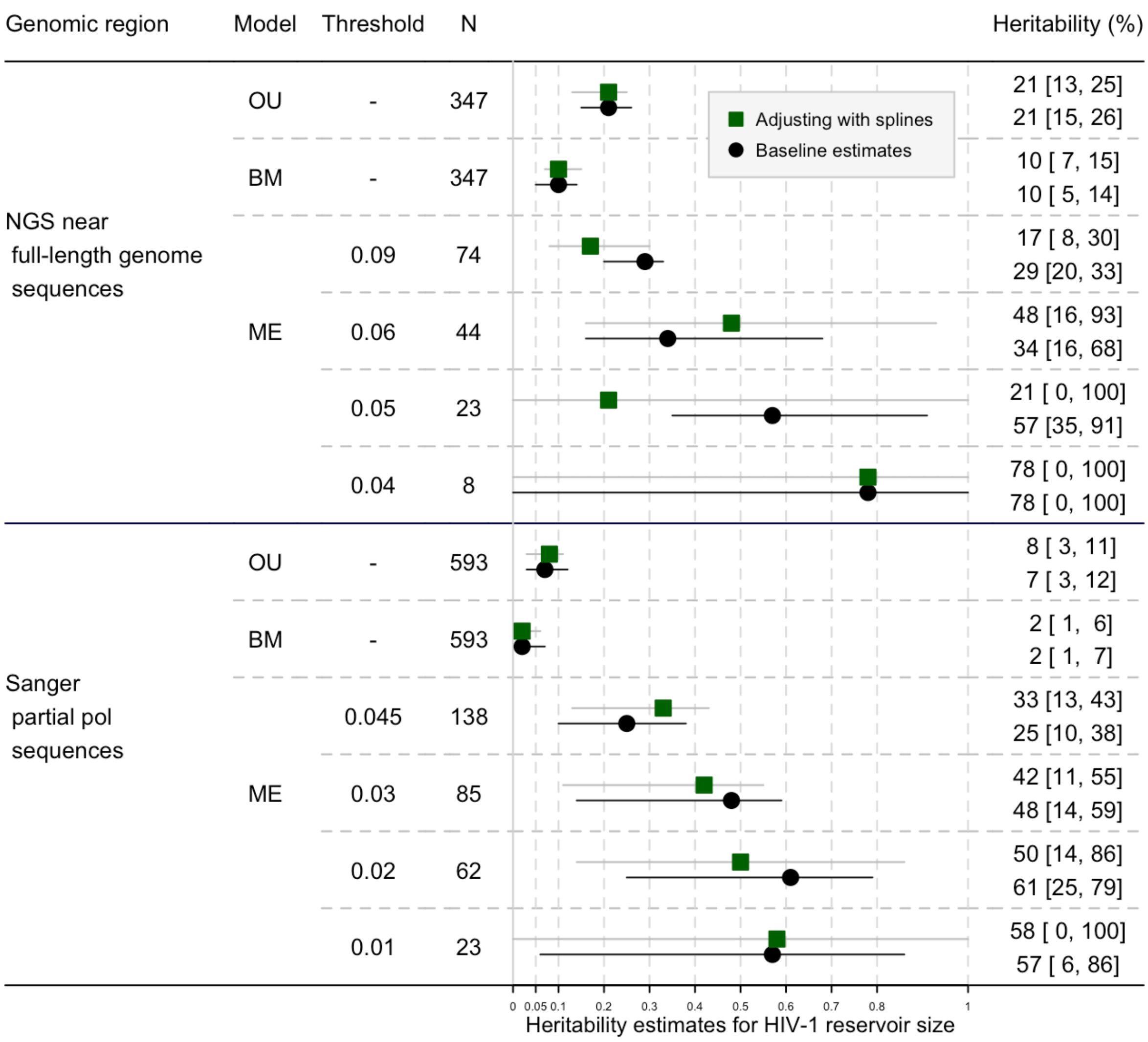

Figure 15. (HIV-1 subtype B only): Adjusted heritability estimates of HIV-1 reservoir size: Sensitivity analysis of adjusting quantitative variables using polynomial splines. 95% confidence intervals are shown in square brackets.

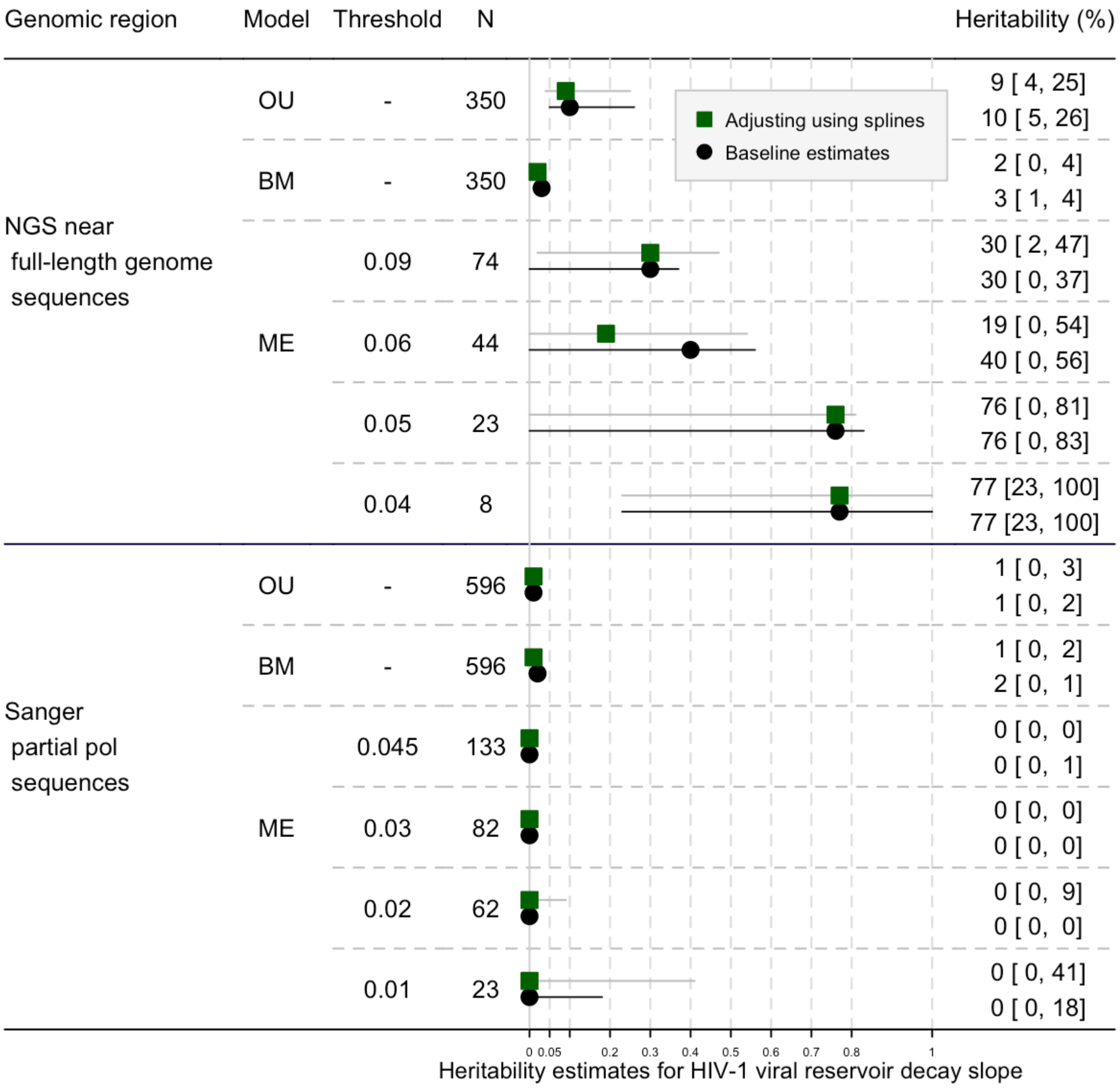

Figure 16. (HIV-1 subtype B only): Adjusted heritability estimates of HIV-1 reservoir decay slope: Sensitivity analysis of adjusting quantitative variables using polynomial splines. 95% confidence intervals are shown in square brackets.

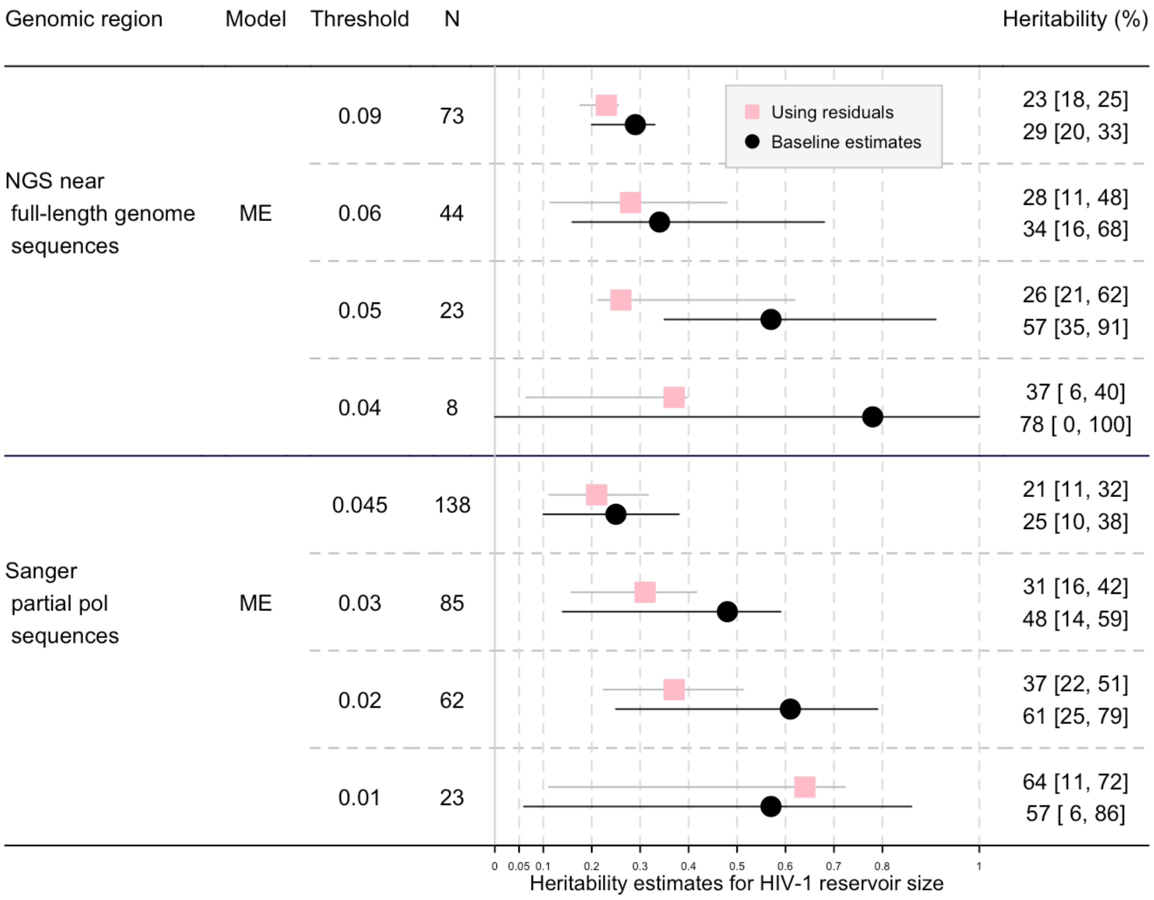

Figure 17. Adjusted heritability estimates of HIV-1 reservoir size: Sensitivity analysis of using residuals of a regression analysis on the full dataset (to get the best information available in the full population for adjustment). 95% confidence intervals are shown in square brackets.

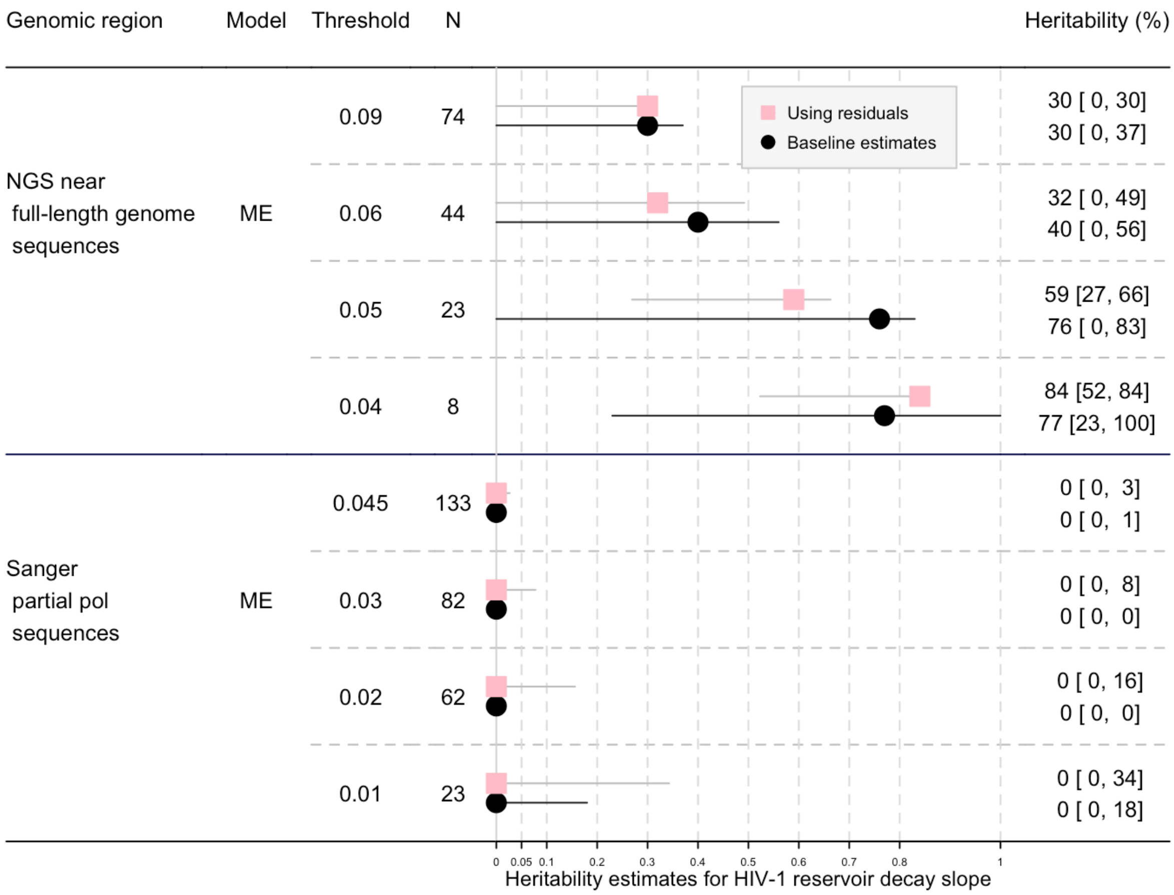

Figure 18. Adjusted heritability estimates of HIV-1 reservoir decay slope: Sensitivity analysis of using residuals of a regression analysis on the full dataset (to get the best information available in the full population for adjustment). 95% confidence intervals are shown in square brackets.

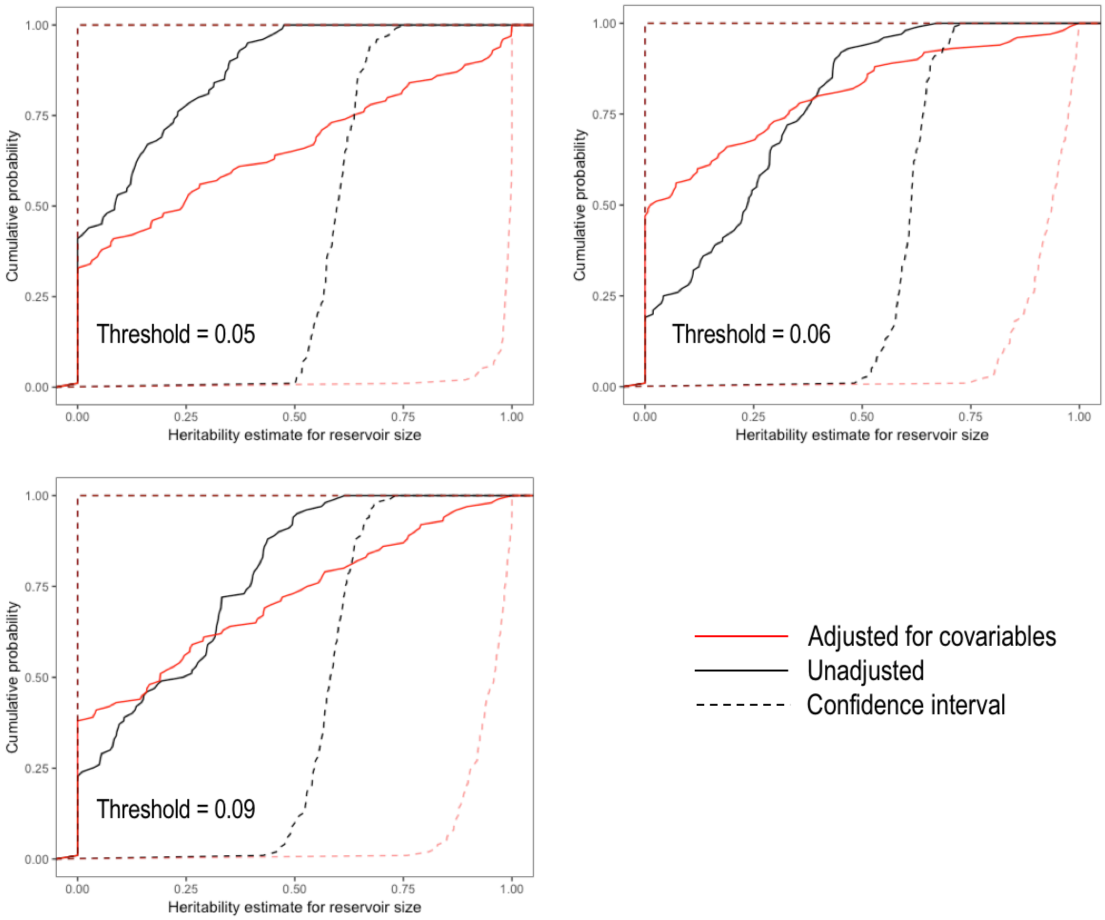

Figure 19. Cumulative probability of heritability estimates for HIV-1 reservoir size with small sample size using mixed-effect model. Under each threshold, 9 clusters were randomly selected from total extracted clusters for 100 times to achieve the accumulative probability. Confidence intervals were calculated respectively from 100 bootstrapped trees.

Supplementary Methods

Statistics

We statistically compared the goodness of fit for four different models: the null model, mixed-effect model, BM model and OU model. The null model was a simple linear regression model with the HIV-1 reservoir size or decay slope as the dependent variable and all potential covariables as the explanatory variables. It regarded patients as different individuals and assumed no correlation between them. In the main analysis, only patients in the transmission clusters were included in mixed-effect model. To make it comparable to other models in terms of sample size, we applied mixed-effect model to the full dataset. Each patient that didn't belong to any transmission clusters was taken as an individual group. By doing so, we didn't change the assumption that correlation was only assumed among patients in the same transmission clusters. For mixed-effect model, loglik was calculated using maximum likelihood estimation. For BM and OU, loglik and logPost was calculated from the R package POUMM[3]. The numbers of degrees of freedom for estimating the unadjusted heritability were 2, 3, 3, 5 for the null model, mixed-effect model, BM and OU model, respectively. The numbers of degrees of freedom for estimating the adjusted heritability of the HIV-1 reservoir size were 17, 18, 18, 20 for the null model, mixed-effect model, BM and OU model, respectively. The numbers of degrees of freedom for estimating the adjusted heritability of the HIV-1 reservoir decay were 13, 14, 14, 16 for the null model, mixed-effect model, BM and OU model, respectively. AIC and BIC for each model were calculated according to loglik and degrees of freedom.

HIV-1 near full-length sequencing

HIV-1 RNA was isolated from 1 ml plasma using the NucleoSpin® RNA Virus Kit (Macherey and Nagel, Oensingen) according to the manufacturer's protocol and an on-column DNAse treatment (3 U DNase (DNase I recombinant, RNase-free; Roche, Mannheim) at RT for 20 min). HIV-1 subtype B sequencing was done according to the protocol published by Di Giallonardo et al. and all other and unknown HIV-1 subtypes were amplified and sequenced following the protocol published by Gall et al.. Briefly, RNA was eluted in 25 µl water separated into two (four – non-B protocol in parenthesis) reverse transcription reactions, each containing 10 (5) µl RNA and a mix of 2 or 3 (1) oligonucleotides for cDNA syntheses (Supplementary Table 10). RNA plus oligonucleotide-mix was incubated at 65°C for 10 min followed by cooling at 4°C for 2 min. cDNA synthesis was performed using the PrimeScript RT (Takara, Saint-Germain-en-Laye) followed by treatment with RNase H (New England Biolabs, Bioconcept, Allschwil) according to the manufacturers' protocol.

Five (Four) PCRs were performed with 2 µl of cDNA using 1 U of Platinum® Taq DNA Polymerase High Fidelity (Invitrogen, Zug),

0.4 mM of each dNTP (Fermentas, Mont-sur-Lausanne), and 0.5 µM oligonucleotides (Supplementary Table xxxxxxx). The PCR cycling conditions were 94°C - 2 min, 35 x (94°C - 30 sec, 55-58°C - 30 sec, 68°C – 2 min 40 sec – 4 min). Amplicons were purified with the Agencourt AMPure XP PCR Purification (Beckman Coulter, Krefeld) according to the manufacturer's instructions and analysed by agarose gel electrophoresis. If required, semi-nested or nested PCRs were performed. Amplicons were quantified using the Quant-iT™ PicoGreen™ dsDNA Assay Kit (Thermofisher Scientific, Reinach) and pooled followed by sequencing library preparation with Nextera XT DNA Sample

Preparation Kit (Illumina, San Diego) according to the manufacturer's protocol. Samples were sequenced using a MiSeq Benchtop Sequencer (Illumina) generating paired-end reads of 2×250 bp length (v2 kit, Illumina).

Table 10. Amplicons and oligonucleotides used for amplification of HIV-1 near full-length genomes.

amplicon ID	oligonucleotide forward		oligonucleotide reverse		oligonucleotide, cDNA synthesis	
	name*	sequence (5'-3')	name*	sequence (5'-3')	name*	sequence (5'-3')
**HIV-1 subtype B**						
A	Ph LTR 455	GGTCTCTCTGGTTAGACCAGATC	pol 3029 rc	GAATATTGCTGGTGATCCTTTCC	RT pol 3043	GTCATGCTACTTTG
A_n	Ph LTR 455	GGTCTCTCTGGTTAGACCAGATC	pol 2787 rc	GTTCTCTGAAATCTACTAATTTTYTCC		
B	gag 2215	CAGGAGCCGATAGACAAGG	int 4778 rc	GTGGATGAATACTGCCATTTGTAC	RT int 4778	GTGGATGAATACTG
B_n	pol 2463	GCTATAGGTACAGTATTAGTAGGACC	int 4564 rc	GTTTTTACTGGCCATCTTCCTGC		
C	pol 3991	CAATTCATCTAGCTTTGCAGGATTCRG	vpu 6110 rc	TTATTGCTACTACTAATGCTACTATTGCT	RT env 6606	TAACACAGAGTGG
C_n	int 4242	ATAGATAAGGCCCAAGAAGAACATG	Ph env 6370 rc	GTTGCTTCTTTCCACACAGG		
D	tat 5783	GGTGTCGACATAGCAGAATAGG	env 8384 rc	GCGGGTCTGAAACGAYAATGGTG	RT env 8423	GATTCCTTCGGG
D_n	tat 6021	CTCATCAAGTTTCTCTATCAAAGCAG	Ph env 8118 rc	CCCACTSCATCCAGGTC		
E	env 7323	GGGGACCCAGAAATTGTAAYGC	Ph LTR R 9635 rc	GAAGCACTCAAGGCAAGC	RT LTR 9636	TGAAGCACTCAAG
E_n	env 7516	GAAAAGCAATGTATGCCCCTCC	Ph LTR R 9635 rc	GAAGCACTCAAGGCAAGC		
**HIV-1 subtype non-B and unknown**						
1	Pan-HIV-1_1F	AGCCYGGGAGCTCTCTG	Pan-HIV-1_1R	CCTCCAATTCCYCCTATCATTTT	Pan-HIV-1_1R	
1_sn	Pan-HIV-1_1F	AGCCYGGGAGCTCTCTG	Pan-HIV-1_1Rn	AYTGTRACDABKGGTCGYTGC		
1_n	Pan-HIV-1_1Fn	GCAGRGAGYTRGAAMGATTYGC	Pan-HIV-1_1Rn	AYTGTRACDABKGGTCGYTGC		
2	Pan-HIV-1_2F	GGGAAGTGAYATAGCWGGAAC	Pan-HIV-1_2R	CTGCCATCTGTTTTCCATARTC	Pan-HIV-1_2R	
2_sn	Pan-HIV-1_2Fn	YTGYTRGTYCAAAATGCRAAYCC	Pan-HIV-1_2R	CTGCCATCTGTTTTCCATARTC		
2_n	Pan-HIV-1_2Fn	YTGYTRGTYCAAAATGCRAAYCC	Pan-HIV-1_2Rn	ACTACTGCCCCTTCACCTTTCC		
3	Pan-HIV-1_3F	TTAAAAGAAAAGGGGGGATTGGG	Pan-HIV-1_3R	TGGCYTGTACCGTCAGCG	Pan-HIV-1_3R	
3_sn	Pan-HIV-1_3F	TTAAAAGAAAAGGGGGGATTGGG	Pan-HIV-1_3Rn	CTCKYCTYYTTGCYYYRGTGGG		
3_n	Pan-HIV-1_3Fn	ATTTTCGGGTTTATTACAGRGACAGCAG	Pan-HIV-1_3Rn	CTCKYCTYYTTGCYYYRGTGGG		
4	Pan-HIV-1_4F	CCTATGGCAGGAAGAAGCG	Pan-HIV-1_4R	CTTWTATGCAGCWTCTGAGGG	Pan-HIV-1_4R	
4_sn	Pan-HIV-1_4Fn	AAAGAGCAGAAGAYAGTGGMAATGARAG	Pan-HIV-1_4R	CTTWTATGCAGCWTCTGAGGG		
4_n	Pan-HIV-1_4Fn	AAAGAGCAGAAGAYAGTGGMAATGARAG	Pan-HIV-1_4Rn	TCMAYTGGTACTAGYTTGWAGCACCA		

*numbers are the start position based on HIV-1_{HXB2} (GenBank accession number K03455)

Reference:

1. Kuznetsova, A., Brockhoff, P. B. & Christensen, R. H. B. lmerTest Package: Tests in Linear Mixed Effects Models. *J. Stat. Softw.* **82**, 1–26 (2017).
2. Zeileis, A. & Hothorn, T. Diagnostic Checking in Regression Relationships. *R News* **2**, 7--10.
3. Mitov, V. & Stadler, T. A Practical Guide to Estimating the Heritability of Pathogen Traits. *Mol. Biol. Evol.* **35**, 756–772 (2018).
4. Hansen, T. F. STABILIZING SELECTION AND THE COMPARATIVE ANALYSIS OF ADAPTATION. *Evol. Int. J. Org. Evol.* **51**, 1341–1351 (1997).
5. Bertels, F. *et al.* Dissecting HIV Virulence: Heritability of Setpoint Viral Load, CD4+ T-Cell Decline, and Per-Parasite Pathogenicity. *Mol. Biol. Evol.* **35**, 27–37 (2018).
6. Blanquart, F. *et al.* Viral genetic variation accounts for a third of variability in HIV-1 set-point viral load in Europe. *PLOS Biol.* **15**, e2001855 (2017).
7. Struck, D., Lawyer, G., Ternes, A.-M., Schmit, J.-C. & Bercoff, D. P. COMET: adaptive context-based modeling for ultrafast HIV-1 subtype identification. *Nucleic Acids Res.* **42**, e144–e144 (2014).
8. To, T.-H., Jung, M., Lycett, S. & Gascuel, O. Fast Dating Using Least-Squares Criteria and Algorithms. *Syst. Biol.* **65**, 82–97 (2016).
