# Supplementary material for: Heritability of the HIV-1 reservoir size and decay under long-term suppressive ART
Source: Nat Commun. 2020 Nov 2;11:5542. doi: 10.1038/s41467-020-19198-7 (PMC7608612; doi:10.1038/s41467-020-19198-7)
Supplement: Supplementary file 3 — Reporting summary [file 41467_2020_19198_MOESM3_ESM.pdf]

nature research

Corresponding author(s): Roger Kouyos

Last updated by author(s): Aug 9, 2020

Reporting Summary

Nature Research wishes to improve the reproducibility of the work that we publish. This form provides structure for consistency and transparency in reporting. For further information on Nature Research policies, see Authors & Referees and the Editorial Policy Checklist .

Statistics

For all statistical analyses, confirm that the following items are present in the figure legend, table legend, main text, or Methods section.

n/a	Confirmed	
☐	☒	The exact sample size (n) for each experimental group/condition, given as a discrete number and unit of measurement
☐	☒	A statement on whether measurements were taken from distinct samples or whether the same sample was measured repeatedly
☐	☒	The statistical test(s) used AND whether they are one- or two-sided<br>*Only common tests should be described solely by name; describe more complex techniques in the Methods section.*
☐	☒	A description of all covariates tested
☐	☒	A description of any assumptions or corrections, such as tests of normality and adjustment for multiple comparisons
☐	☒	A full description of the statistical parameters including central tendency (e.g. means) or other basic estimates (e.g. regression coefficient) AND variation (e.g. standard deviation) or associated estimates of uncertainty (e.g. confidence intervals)
☐	☒	For null hypothesis testing, the test statistic (e.g. F, t, r) with confidence intervals, effect sizes, degrees of freedom and P value noted<br>*Give P values as exact values whenever suitable.*
☐	☒	For Bayesian analysis, information on the choice of priors and Markov chain Monte Carlo settings
☐	☒	For hierarchical and complex designs, identification of the appropriate level for tests and full reporting of outcomes
☒	☐	Estimates of effect sizes (e.g. Cohen's d, Pearson's r), indicating how they were calculated

Our web collection on statistics for biologists contains articles on many of the points above.

Software and code

Policy information about availability of computer code

Data collection	All data was collected on an OpenBIS database.
Data analysis	All data was analyzed using R version 3.5.1. R packages used include APE, NLME, POUMM and splines. All R code can be made available upon request. NGS sequences were preprocessed with PRINSEQ v0.20.4. We aligned the preprocessed sequences to an HXB2 reference genome and generated the consensus sequences with a majority vote rule using ngshmmalign from V-pipe. Phylogenetic tree constructions were performed using the maximum likelihood algorithm RAxML version 8 with the GTRCAT model. To avoid the risk brought by rooting with distant outgroup, we additionally performed a sensitivity analysis using LSD-0.2 to find the root of the tree. HIV-1 subtype was determined using the REGA HIV-1 subtyping tool and COMET.

For manuscripts utilizing custom algorithms or software that are central to the research but not yet described in published literature, software must be made available to editors/reviewers. We strongly encourage code deposition in a community repository (e.g. GitHub). See the Nature Research guidelines for submitting code & software for further information.

Data

Policy information about availability of data

All manuscripts must include a data availability statement. This statement should provide the following information, where applicable:

- Accession codes, unique identifiers, or web links for publicly available datasets
- A list of figures that have associated raw data
- A description of any restrictions on data availability

The datasets generated during and/or analyzed during the current study are not publicly available due to privacy reasons, the sensitivities associated with HIV infections, and the representativeness of the dataset. We consider a publication of the nucleotide sequences as too problematic because they would allow reconstructing transmission events and can serve as patient-identifiers allowing to link the very rich epidemiological and virological information to other datasets in an uncontrolled way (and thereby jeopardizing patient privacy). All data in the SHCS, including viral genomes, can be used for well-defined projects that are in accordance with the guidelines of the SHCS, if a corresponding project proposal is approved by the SHCS scientific board (www.shcs.ch).

Field-specific reporting

Please select the one below that is the best fit for your research. If you are not sure, read the appropriate sections before making your selection.

- [x] Life sciences
- [ ] Behavioural & social sciences
- [ ] Ecological, evolutionary & environmental sciences

For a reference copy of the document with all sections, see nature.com/documents/nr-reporting-summary-flat.pdf

Life sciences study design

All studies must disclose on these points even when the disclosure is negative.

Sample size	Our analyses are based on a longitudinal, observational cohort study (www.shcs.ch).We did not perform sample size calculations, but rather included all SHCS participants fulfilling the inclusion criteria described in the Methods section.
Data exclusions	As recommended for analyses based on observational cohort studies, we followed STrengthening the Reporting of OBservational studies in Epidemiology (STROBE) criteria as shown in figure 1 in detail. 1932 individuals were selected from 18688 individuals enrolled in Swiss HIV Cohort Study according to the following criteria: HIV-1 infection; receiving ART for >=5 years; no treatment interruption of >7 days; no virological failure defined as two consecutive measurements >200 HIV-1 RNA copies/ml plasma; PBMC available for three time points: 1.5+/- 0.5 years, 3.5 +/- 0.5 years, and 5.5 +/- 1 years after initiating first-line ART; 4th PBMC sample, latest time point available if receiving ART for >=8 years (optional). Further 550 individuals were excluded for starting on less potent ART regimens, i.e., mono- or dual therapy, less potent/ unboosted PI (NFV, SQV etc.). 1166 individuals were further selected for having >=3 PBMC samples (mandatory 1st - 3nd time point). 109 were excluded for not having successful total HIV-1 DNA quantification in >=3 PBMC samples (mandatory 1st - 3nd time point). Of the 1057 individuals left, 475 individuals with available NGS sequencing of the near full-length genome from plasma at the latest sample before the initiation of ART were denoted as population A0 and 869 individuals with available Sanger sequencing of partial pol region obtained for genotypic resistance testing were denoted as population B0. Of the two populations, individuals infected with HIV-1 subtype B were finalized as population A and B, which were our main study population in the paper.
Replication	Replicates of patients' samples were not performed due to the limited number of available cells stored in the SHCS biobank. Multiple controls were included in the droplet digital PCR assays to ensure reproducibility.
Randomization	This study was an observational study and not randomized. However, it relies on data of the Swiss HIV cohort study, which collects a large number of variables. Thus, we were able to correct for many known or suspected confounders. For HIV-1 reservoir size, we adjusted for transmission group, sex, ethnicity, age, time on ART, time to suppression, initiation of ART in acute/chronic infection, RNA and CD4 pre-ART, and prior viral blips. For reservoir decay slope, we adjusted for HIV-1 reservoir size, treatment center, time to suppression, CD4 pre-ART and viral blips.
Blinding	Personnel who measured total HIV-1 DNA were blinded in terms of patients from whom the samples were derived and their respective covariables studied. Investigators were also blinded regarding patient information from the Swiss HIV Cohort Database: patients are annonymized in the SHCS database.

Reporting for specific materials, systems and methods

We require information from authors about some types of materials, experimental systems and methods used in many studies. Here, indicate whether each material, system or method listed is relevant to your study. If you are not sure if a list item applies to your research, read the appropriate section before selecting a response.

Materials & experimental systems

n/a	Involved in the study	
x		Antibodies
x		Eukaryotic cell lines
x		Palaeontology
x		Animals and other organisms
	x	Human research participants
x		Clinical data

Methods

n/a	Involved in the study	
x		ChIP-seq
x		Flow cytometry
x		MRI-based neuroimaging

Human research participants

Policy information about studies involving human research participants

Population characteristics	We included 1,057 HIV-infected adults enrolled in the SHCS. Their characteristics are summarized in detail in Table 1 of the manuscript. Systematic selection of these very well characterized and longitudinally studied patients within the SHCS is a key strength of this study.
Recruitment	There was no recruitment for our study. We used biobanked samples and data collected from the pre-existing longitudinal Swiss HIV cohort study as detailed in the methods section. The sole inclusion criteria for participating in the SHCS is to be above the age of 16 and to be HIV-infected. It is an open cohort that continuously recruits individuals from all transmission groups.
Ethics oversight	The SHCS was approved by the ethics committees of the participating institutions (Kantonale Ethikkommission Bern, Ethikkommission des Kantons St. Gallen, Comite Departemental d'Ethique des Specialites Medicales et de Medicine Communataire et de Premier Recours, Kantonale Ethikkommission Zürich, Repubblica et Cantone Ticino–Comitato Ethico Cantonale, Commis- sion Cantonale d'Etique de la Recherche sur l'Être Humain, Ethikkommission beider Basel for the SHCS and Kantonale Ethikkommission Zürich for the ZPHI), and written informed consent was obtained from all participants.

Note that full information on the approval of the study protocol must also be provided in the manuscript.
